# Proteomic comparison by iTRAQ combined with mass spectrometry of egg white proteins in laying hens (*Gallus gallus*) fed with soybean meal and cottonseed meal

**DOI:** 10.1371/journal.pone.0182886

**Published:** 2017-08-15

**Authors:** Tao He, Haijun Zhang, Jing Wang, Shugeng Wu, Hongyuan Yue, Guanghai Qi

**Affiliations:** Key Laboratory of Feed Biotechnology of Ministry of Agriculture, Feed Research Institute, Chinese Academy of Agricultural Sciences, Beijing, China; Leibniz-Institut fur Pflanzengenetik und Kulturpflanzenforschung Gatersleben, GERMANY

## Abstract

Cottonseed meal (CSM) is commonly used in hens’ diets to replace soybean meal (SBM). However, the molecular consequences of this substitution remains unclear. To investigate the impact of this substitution at the molecular level, iTRAQ combined with biochemical analysis was performed in Hy-Line W-36 hens supplemented with a mixed diet of CSM and SBM. Egg weight, albumen height, and Haugh unit were significantly reduced in the CSM_100_ group (100% crude protein of SBM replaced by CSM) compared with the SBM group (*P*<0.05). A total of 15 proteins, accounting for 75% of egg white proteins with various biological functions of egg whites, were found to be reduced. This finding may relate to the decrease of albumen quality in the CSM_100_ group. Oviduct magnum morphology and hormone analysis indicated that a reduced level of plasma progesterone caused reduced growth of the tubular gland and epithelial cells in the magnum, further decreasing egg white protein synthesis in the magnum. These findings help demonstrate the molecular mechanisms of a CSM diet that cause adverse effects on albumen quality, while also showing that SBM should not be totally replaced with CSM in a hen diet.

## Introduction

Egg white, or albumen, is an important food material that provides many essential nutrients to human. Egg white proteins are the main components of egg whites, constituting 10–12% of the protein content, and are secreted by the magnum of the oviduct where all the egg white components are produced. Egg white proteins are composed mainly of ovalbumin, ovotransferrin, ovomucoid, ovomucin, lysozyme, ovoinhibitor, and avidin. Ovalbumin represents more than 50% of total proteins, followed by ovotransferrin with 11–13%. These egg white proteins are associated with diverse biological properties, including antimicrobial activity, protease inhibition, and immunologic characteristics [1].

Soybean meal (**SBM**) is currently the most commonly used plant protein ingredient in poultry feed and comprises up to 50% of the poultry diet. However, a shortage of SBM supply and the increase of SBM price prevents higher use in the poultry diets. Replacement of SBM with less expensive plant protein sources would be beneficial for reducing feed costs and saving protein resources in the animal feed industry. Cottonseed meal (**CSM**), a byproduct of the process of oil extraction from cotton seeds, is an attractive alternative protein ingredient for poultry diets [2]. However, CSM has a consistently lower feeding value, attributable to free gossypol (**FG,** a main anti-nutritional factor of CSM), with lower yields of energy and lysine compared to SBM. Numerous studies have proven that a CSM diet was associated with negative performance and lower albumen quality of fresh or stored eggs in poultry [2]. Egg weight was decreased from hens fed a diet with 150 g/kg CSM in fresh eggs [3], and in a diet with 100 g/kg CSM in stored eggs [4]. Haugh unit (**HU**) and albumen height were significantly reduced from hens fed a diet with 100 g/kg CSM in fresh eggs [5] or in eggs stored at room temperature compared with SBM diets [4]. Albumen height and HU are important factors for evaluating albumen quality. However, previous studies on CSM effects were analyzed by traditional physiological and biochemical methods, and there is no research focused on the molecular mechanisms of albumen quality reduction induced by CSM diets.

Currently, little research can be found for application of proteomic methods on hen egg whites [1]. Linear ion trap Orbitrap and peptide ligand libraries were applied for giving a comprehensive view of egg white, and 148, 158 proteins were identified, respectively[6,7]. Thirty-two protein spots representing 8 proteins were identified with significant differences in abundance when stored at different temperatures using two-dimensional electrophoresis(**2-DE**) followed by MALDI-TOF MS/MS[8]. However, these result was far from the whole protein species in the egg white because of the wide range molecular masses, isoelectric point values, and the concentration varying greatly from one protein to another. Consequently, some minor proteins are unable to be detected easily. High-throughput methods were needed for the separation and detection of egg white proteins. Isobaric tags for the relative and absolute quantification labeling technique (**iTRAQ**) has a higher sensitivity compared with 2-DE and isotope coded affinity tags (**ICAT**) [9,10], and can be applied to identify and quantify egg white protein expression levels and identify small changes in protein expression [11]. Presently, iTRAQ reagents have not been used in egg white protein studies for hens fed with different diets. The purpose of this study is to identify the different biochemical changes that result from changing feed ratios between CSM and SBM and to evaluate egg white proteins on a proteomic level. This will help demonstrate molecular-level protein differences in egg whites and may help promote better feeding choices in animal feed.

## Material and methods

### Experimental design and diets

This study was approved by the Animal Care and Use Committee of the Feed Research Institute of the Chinese Academy of Agricultural Sciences. A total of 216 40-wk-old Hy-line W36 laying hens were given 3 dietary treatments with six replicates of 12 birds each. The control group was fed a corn and soybean meal basal diet, and the other two experimental diets were with 50% (**CSM**_**50**_) and 100% (**CSM**_**100**_) of the dietary protein content provided by SBM replaced by CSM. The feeding trial lasted for 12 weeks. The ingredients and nutrient composition of experimental diets are given in **Table 1.** All groups had similar ratios between different essential amino acids in relation to lysine through supplemented crystalline AA, with a fixed dietary protein of 16% and an energy concentration of metabolizable energy (ME) of 11.08 MJ /kg feed. Hens were maintained on a 16-h light schedule and allowed ad libitum access to experimental diets and water. Room temperature was maintained at 15 ± 2°C. The diets were fed in mash form (water: feed, V/V = 2:1) during the entire experimental period.

**Table 1:** Composition and nutrient levels of laying hen’s diets from 40 to 51 wk of age.

Items	Treatment^1^			Items	Treatment^1^		
	SBM(control)	CSM_50_	CSM_100_		SBM(control)	CSM_50_	CSM_100_
Ingredient, %				Nutrient levels^3^,%			
Corn	63.37	64.3	65.30	ME,MJ/kg	11.09	11.08	11.09
Soybean meal	22.91	10.85	0	Crude protein^4^	16.13	16.07	16.08
Cottonseed meal	0	9.83	18.9	Calcium^5^	3.48	3.52	3.46
Calcium phosphate	1.48	1.47	1.38	Availablephosphorus	0.35	0.35	0.34
Limestone	8.55	8.58	8.54	Lysine	0.813	0.813	0.813
NaCl	0.30	0.30	0.30	Methionine	0.408	0.407	0.407
DL-Methionine	0.179	0.252	0.204	Methionine+cysteine	0.729	0.730	0.728
Lysinel-HCl	0.107	0.328	0.523	Tryptophan	0.171	0.171	0.171
L-Threonine	0.102	0.177	0.243	Threonine	0.576	0.575	0.576
L-Valine	0.057	0.125	0.182	Cysteine	0.325	0.323	0.323
L-Isoleucine	0.060	0.177	0.278	Valine	0.712	0.711	0.712
L-Tryptophan	0	0.013	0.022	Isoleucine	0.652	0.651	0.650
L-Cysteine	0.160	0.237	0.153	Leucine	1.269	1.108	0.971
Premix^2^	0.23	0.23	0.23	Arginine	0.921	1.071	1.210
Phytase enzyme	0.05	0.05	0.05	FG, mg/kg^6^	0	17.45	20.16
Cholinechloride (50%)	0.10	0.1	0.10				
Zeolite	2.345	2.981	3.595				
Total	100	100	100				

^1^ CSM, cottonseed meal; the CSM_50_, CSM_100_ represent 50%,100% dietary protein contents provided by soybean meal were replaced by CSM, correspondingly 98.3g, 189 g/kg CSM were added in the diets. Control group is corn-soybean meal (SBM) basal diet.

^2^ Provided per kg ofdiet: vitamin A, 12,500 IU; vitamin D_3_, 2500 IU; vitamin E, 15 IU; vitamin K_3_, 2.65 mg; vitamin B_1_, 2 mg; vitamin B_2_, 6 mg; vitamin B_12_, 0.025 mg; nicotinic acid, 50 mg; calcium pantothenate, 12 mg; biotin, 0.0325 mg; folic acid, 1.25 mg; iron, 80 mg; copper, 8 mg; manganese, 100 mg; zinc, 75 mg; iodine, 0.35 mg; selenium, 0.15 mg.

^3^ Calculation based on digestible amino acid.

^4,5^ ^6^Analyzed values.

### Performance, albumen quality and sample collection

Performance and albumen quality parameters were evaluated during the feeding trial of 40–51 weeks. The mean body weights of laying hens were 1.76 ± 0.2 kg at the beginning of the trial. Feed intake and feed conversion ratio (FCR, feed/egg, g/g) were measured every two weeks, and egg production, egg weight and mortality were recorded daily.

Five eggs were collected from each replicate at the end of the feeding trial to determine albumen quality indices. The albumen height and HU were determined with Egg Analyzer (ORKA Food Technology Ltd, Ramat Hasharon, Israel). All results were obtained at room temperature.

At the end of week 51, one laying hen from each replicate was randomly selected. Blood was collected via wing vein and centrifuged at 3000 × g for 10 min to harvest serum, which was then stored at –20°C until analysis. Layers were immediately sacrificed by cervical dislocation. The magnam of oviduct were excised and fixed in 10% buffered neutral formalin (Sinopharm Chemical Reagent Beijing Co., Ltd., Beijing, China), respectively.

### Oviduct magnum morphology assessment and hormone analysis

For histopathological studies, the formalin-fixed oviduct (magnum) samples were stained with hematoxylin and eosin (HE). All reagents used were analytical grade (Sinopharm Chemical Reagent Beijing Co., Ltd. Beijing, China). The histopathological changes were examined with a light microscope (BX51, Olympus Corp., Tokyo, Japan). Histopathological assessment was performed by qualified staff at the Department of Veterinary Pathology in the Beijing University of Agriculture. Plasma progesterone and estrogen concentrations were analyzed using iodine [125I] estradiol and a progesterone radioimmunoassay kit (Beijing North Institute of Biological Technology, Beijing, China).

### Trypsin digestion and iTRAQ labeling

All the reagents and buffers needed for iTRAQ labeling and cleaning were purchased from Applied Biosystems (Foster City, CA). The iTRAQ labeling protocol was performed according to the manufacturer’s instructions [9]. In brief, egg white from the CSM_100_ and SBM group was dissolved by 8 M urea supplemented with 10 mM DTT, pH 8.5 (Amesco St. Louis, MO), and total protein contents was determined by the Bradford assay. Proteins were dissolved, denatured, alkylated and digested with trypsin (Promega) at 37°C overnight. To label peptides with the iTRAQ reagent, 1 unit of label (defined as the amount of reagent required to label 100μg protein) was thawed and reconstituted in 150μL of isopropanol. The digestions from CSM_100_ and SBM were labeled with 114 and 116 iTRAQ reagents, respectively, and this reaction was repeated with 115 and 117 iTRAQ reagents to guarantee the accuracy of quantitation. Moreover, a strong cation exchange (SCX) column (Applied Biosystems) was used for separating the mixed peptides. The elution buffer was used as following: elution buffer A containing 5mM K_2_HPO_4_ in 20% (v/v) acetonitrile, at pH 3.0 while elution buffer B containing 5 mM K_2_HPO_4_ in 20% (v/v) acetonitrile, 350 mM KCl at pH 3.0. The labeled peptides were reconstituted in phase A and injected at a flow rate of 0.7ml/min onto a high resolution SCX column (4.6×250mm 5μm; ThermoBioBasi). The labeled peptides mixture was diluted by buffer A and the pH of diluted sample mixture was adjusted to 3.0 with phosphoric acid. The diluted sample mixture was loaded onto the cation exchange cartridge, and the SCX column and C18 precolumn were flushed with a 3-step gradient potassium chloride solution (0, 50 and 100 mM) for 66 min. In total, 10 fractions were collected during SCX fractionation step, and all the fractions were desalted with SP-10 precolumn on an Agilent 1100 series HPLC system with 5% (v/v) acetonitrile. Each collected fraction was concentrated by vacuum centrifugation and reconstituted in 20μL of HPLC load buffer prior to microcapillary LC-ESI MS/MS analysis.

### Analysis by Q-Exactive mass spectrometer and data processing

All fractions eluted from SCX were analyzed by a Q-Exactive mass spectrometer fitted with a nano-liquid chromatography system. The eluent was introduced directly to a Q-Exactive mass spectrometer via EASY-Spray ion source. The on-line nano-liquid chromatography system (Thermo Scientific EASY-nLC 1000 System) with 12cm capillary columns of an internal diameter of 75 μm filled with 3 μm Reprosil-Pur C18 resin was used for peptide separation. Peptides were eluted by using a binary solvent system with 99.9% H_2_O, 0.1% formic acid (phase A) and 99.9% ACN,0.1% formic acid (phase B). The following linear gradient was used: 4–8% B in 5 min, 8–35% B in 35 min, 35–90% B in 5 min, washed at 95% B for 6 min, and equilibrated with 4% B for 8 min at a 350 nL/min flow rate. The eluent was introduced directly to a Q-Exactive mass spectrometer via EASY-Spray ion source(Thermo Fisher Scientific, Waltham, MA, USA). Source ionization parameters were as follows: spray voltage, 2.1 kV; capillary temperature, 250°C; and declustering potential, 100 V. The mass spectrometer was operated in a Top 20 data-dependent mode with automatic switching between MS and MS/MS. Full-scan MS mode (350−1800 m/z) was operated at a resolution of 70 000 with automatic gain control (AGC) target of 1 × 10^6^ ions and a maximum ion injection time (IT) of 60 ms. The precursor ions are fragmented by high-energy collisional dissociation (HCD) and subjected to MS/MS scans with the following parameters: resolution, 17 500; AGC, 5 × 106 ions; maximum IT, 70 ms; intensity threshold, 5000; and normalized collision energy, 29%.

### Data interpretation and quantitation

For data processing, we used Proteome Discoverer software 1.2 (Thermo Fisher Scientific, Waltham, MA, USA) to interpret raw data files produced by mass spectrometry. The CHICK protein database was downloaded from NCBI database and combined with the reversed sequences and sequences of widely spread contaminants, such as human keratins. The parameters for database searching were as following: trypsin was selected as the enzyme; two missed cleavages were allowed at maximum; precursor mass tolerance was set to 15 ppm; fragment mass tolerance was set to 20 mmu; carbamidomethylation of cysteine was set as fixed modification; methionine oxidation and iTRAQ 4plex labels at the N-termini and at lysine side chains were allowed as dynamic modification. Strict maximum parsimony principle was applied andonly peptide spectrum matches (PSMs) with high or medium confidence and with delta Cn better than 0.15 were considered for protein grouping. Ion peaks were integrated based on the most confident centroid with 20 ppm tolerance. By reverse database, the false discovery rate was set to 0.01 to get high confidence result.

Protein quantification was also performed by Proteome Discoverer software 1.2, which automatically calculated the relative abundance of iTRAQ-labeled peptides and the corresponding proteins. Only the unique and high confident peptides were considered for quantification. To correct the bias of pipetting or the determination of protein concentration in the mixed samples, all protein ratios were normalized by the median protein ratio. The minimum significant regulation thresholds was 1.2. The proteins with ratios ≥1.2 or≤0.833 are considered as the differentially expressed proteins.

### Western blotting

The protein concentration of the supernatant was measured by the Bradford protein assay. Samples containing 30μg of total protein were separated by 12% (w/v) SDS-PAGE and transferred onto a PVDF membrane (Millipore, Bedford, MA). After incubating for 1 h with blocking buffer (5% (w/v) nonfat milk in TBS-T (0.05% (v/v) Tween 20 in Tris-buffered saline), the membrane was probed with the indicated primary antibodies diluted in blocking buffer overnight at 4°C. After being extensively washed with TBS-T, the membrane was incubated with horseradish peroxidase-conjugated antibody to rabbit (Cell Signaling Technology) diluted in blocking buffer (1:2000) for 1 h at room temperature. Bands were visualized with Super Signal West Pico Chemiluminescent Substrate (Pierce) and recorded on x-ray films (Fuji Medical, Tokyo, Japan). Finally, the visualized bands were quantified by QUANTITY ONE software on a GS-800 densitometer (Bio-Rad). The antibodies including anti-Ovoinhibitor (ab193507), anti-Lysozyme (ab391) and anti-Avidin (ab6675), were purchased from Abcam company (Cambridge, MA, US).

### Statistical analysis

All data were analyzed using the one-way analysis of variance (ANOVA), and means were compared by the Duncan’s multiple range test (SAS Institute, 2001). Results were presented as means ± SEM. Effects were considered significant when P<0.05.

## Results

### Performance and albumen quality

No hens from any group displayed signs of clinical illness during the feeding period. The inclusion of CSM had an adverse effect on the performance of laying hens and albumen quality (Table 2). There was no significant difference between the CSM_50_ and control groups. However, laying hens fed diets of CSM_100_ had lower feed intake and egg weight compared with the control (P<0.05). HU, albumen height and albumen weight were significantly reduced in the CSM_100_ diet (P<0.05, Table 2).

**Table 2:** The performance, albumen quality and concentrations of plasma progesterone and estrogen in hens fed diets formulated with soybean meal (SBM) and diets that replaced 50% or 100% crude protein content of SBM with cottonseed meal (CSM_50_ or CSM_100_) at 51 weeks of age.

	SBM	CSM_50_	CSM_100_	SEM	ANOVA
Feed intake, g/hen∙d	126.29^a^	123.32^a^^b^	117.93^b^	1.297	0.017
Egg production, %	89.90	90.06	88.19	0.684	0.493
Egg weight, g/egg	65.44^a^	65.10^a^	62.83^b^	0.354	0.002
FCR, % ^1^	2.07	2.03	2.07	0.013	0.396
Albumen height, mm	6.15^a^	5.89^a^	5.20^b^	0.130	0.002
Haugh unit	70.45^a^	69.48^a^^b^	68.11^b^	0.421	0.020
Albumen weight	40.67^a^	41.04^a^	38.72^b^	0.409	0.033
Progesterone, ng/mL	0.064^a^	0.067^a^	0.048^b^	0.003	0.036
Estrogen, pg/mL	989.96	1022.10	885.69	30.203	0.157

^ab^Means in the same row with different superscripts differ significantly (P<0.05). Values are presented as means with SEM (n = 6).

^1^ FCR, feed conversion ratio

### Histological changes and plasma hormone concentration

**Fig 1:**
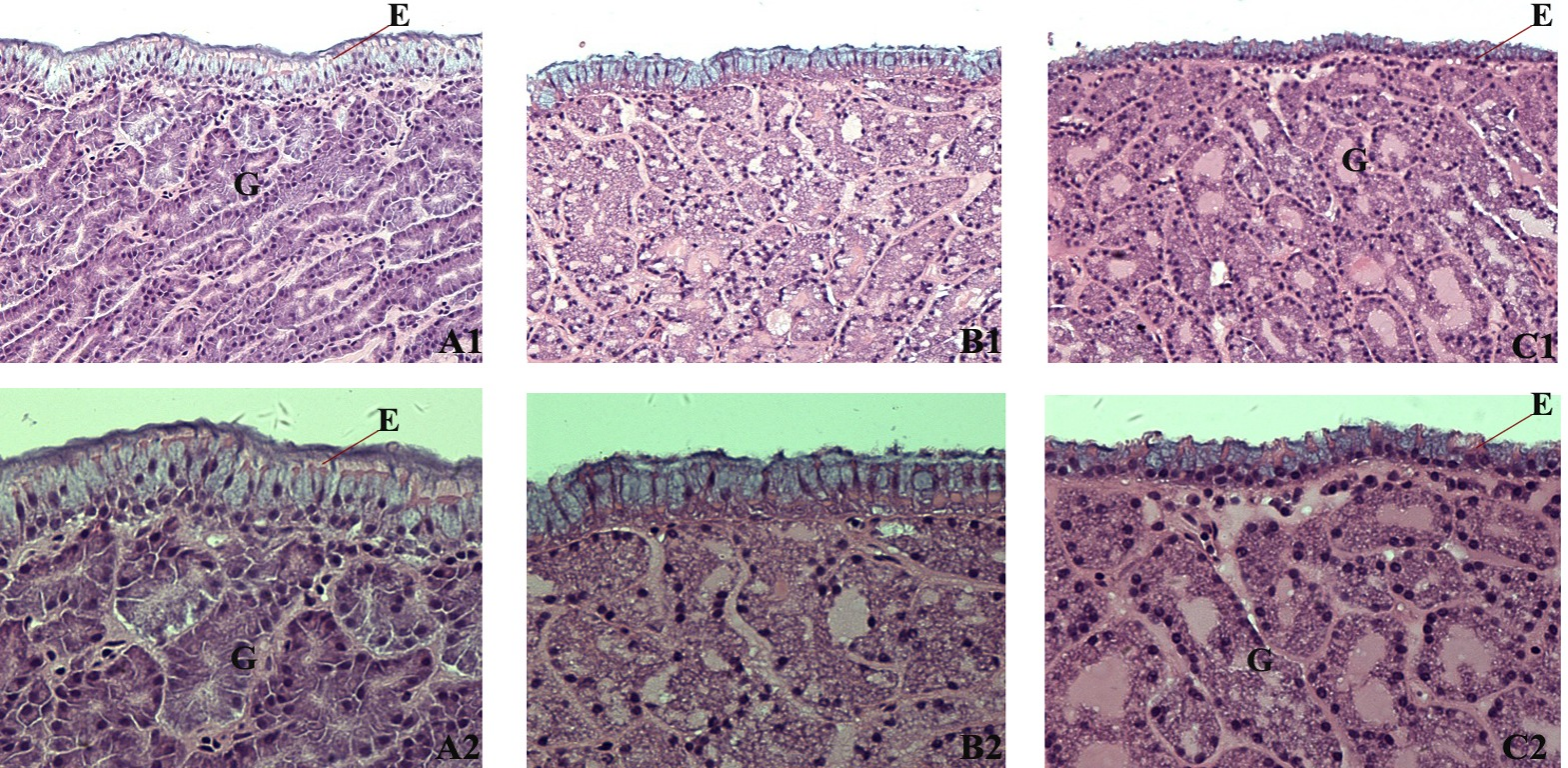
Oviduct magnum histopathology of laying hens fed diets formulated with soybean meal (SBM) and diets that replaced 50% or 100% crude protein content of SBM with cottonseed meal (CSM_50_ or CSM_100_) at 51 weeks of age. (HE staining. A, B, and C represent the SBM, CSM_50_ and CSM_100_ groups, respectively. A1, B1 and C1 magnification 200×; A2, B2 and C2 magnification 400×) G: tubular gland cell; E: epithelium.

Histological lesions were not observed in the oviduct tissues of hens among all groups. A depressed growth of tubular gland and epithelial cells was observed with increasing CSM supplementation, especially in the CSM_100_ group (Fig 1). There was no significant difference in plasma progesterone and estrogen levels between the CSM_50_ and SBM groups, whereas the progesterone level was significantly decreased in the CSM_100_ group (P<0.05) (Table 2).

Tubular gland cells were larger in the SBM group than in the CSM_100_ group, and the secretory granules near the apex of most of the epithelial cells were generally smaller in the CSM_100_ group and contained a less flocculent material, either filamentous or granular. Fewer ciliated cells were also observed in the epithelial layer in the CSM_100_ group.

### Protein identification, quantitation and analysis

With high through-put mass spectrometry, 95 proteins were definitively identified from egg white by 12813 mass spectrums (*Details in S1 Table*). Both high abundance proteins including ovalbumin, ovotransferrin, ovomucin, ovoinhibtor, lysozme, and low abundance proteins including CEPU-Se, tenascin, fibrinogen and di-N-acetylchitobiase, *etc*. were detected in the egg white. Meanwhile, 39 identified proteins were overlapped compared with the result in the two previous studies[6,7], such as hen lysozyme, ovomucoid, and angiopoietin-related protein.

**Fig 2:**
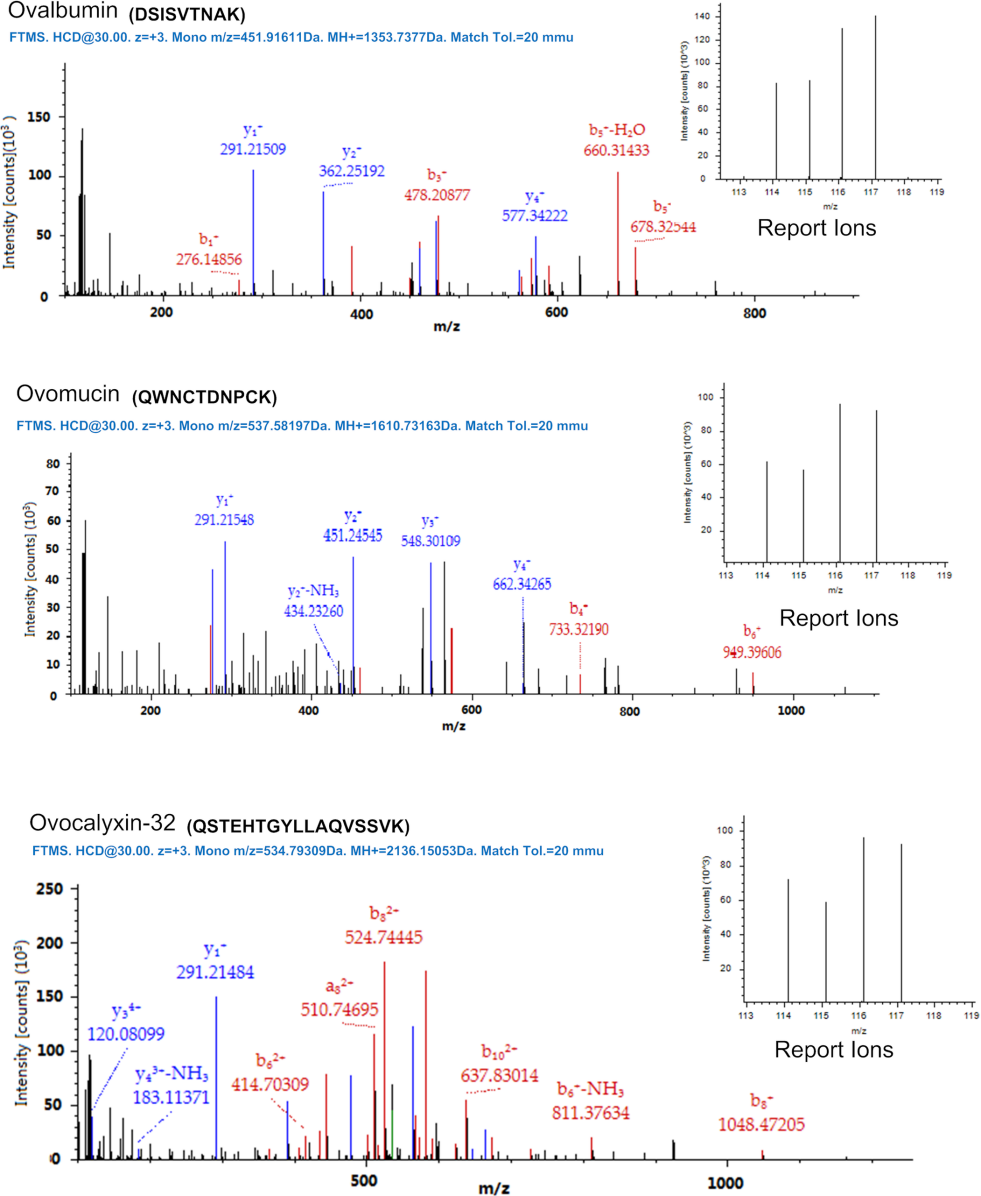
The mass spectra and reporter ion results for ovalbumin, ovomucin and ovocalyxin-32. The proteins were analyzed according to the y and b ions, such as y1, y2, y3 and y4 in ovomucin. The quantitative data were calculated from the peaks obtained for reporter ions that are shown on the right side.

According to iTRAQ tags, 17 differentially expressed proteins that changed by more than 1.2-fold were observed between the egg white from hens fed the CSM_100_ diet and those fed the SBM diet (Table 3). Combined with results from iTRAQ tools, we obtained an accurate quantitative result by reporter ions (Fig 2). Compared to the SBM group, 15 proteins were down-regulated, while 2 proteins were up-regulated in the CSM_100_ group. To better understand the differentially expressed proteins between the SBM and CSM100 groups, the Uniprot database and Gene Ontology [12] were used for the annotation of 17 proteins. The results showed the 15 reduced proteins were involved in various biological processes and functions, such as anti-bacterium or anti-virus activity, metabolic process, immune system process, biological adhesion, response to stimulus, reproduction, biological regulation. For example, Ig J polypeptide and prothrombin precursor play an important role in immune system process, response to stimulus. Ovomucin precursor plays a significant role in biological adhesion. Ovalbumin and ovomucin are important to the viscocity and gel property of egg white (Table 3). The up-regulated 2 proteins were related with cellular component organization or biogenesis.

**Table 3:** The significantly altered egg white proteins of laying hens fed diets formulated with soybean meal (SBM) and diets that replaced the 100% crude protein content of SBM with cottonseed meal (CSM_100_).

GI_Accession No^1^	Description	Peptides^2^	SBM/CSM_100_^3^	SBM/CSM_100_^4^	MW [kDa]^5^	calc. pI^6^
440923751	ovalbumin	25	1.455	1.480	42.87	5.29
6729945	ovotransferrin	29	1.403	1.460	36.17	7.06
229157	lysozyme	12	1.509	1.428	14.30	8.85
45382809	ovomucin precursor	64	1.598	1.682	233.30	5.60
45382467	clusterin precursor	15	2.097	2.248	51.31	5.66
762068625	ovoinhibitor precursor	17	1.843	2.102	51.85	6.58
257357678	ovocalyxin-32	2	1.298	1.581	30.75	9.08
127513	Ig mu chain C region	4	1.347	1.337	48.14	6.41
46049078	Ig J polypeptide	1	1.737	1.285	17.93	5.35
513221142	predicted: platelet-activating factor acetylhydrolase 2	1	1.598	1.483	34.61	8.57
1536812	Ig heavy chain variable region	1	1.536	1.832	10.80	5.31
45382957	prothrombin precursor	1	1.450	1.435	69.06	5.65
746815865	ovoDB1	1	1.250	1.386	7.10	9.56
558704994	avidin	3	1.873	1.605	14.29	9.59
5705960	Ig alpha heavy chain	3	2.207	2.226	61.46	4.71
211055	beta-actin	1	0.621	0.608	9.34	5.12
363733143	predicted:carboxypeptidase E	2	0.810	0.710	52.36	5.23

^1^ Accession numbers of differentially expressed proteins according to the NCBInr database.

^2^ The matched peptides correspond to proteins. Both the sequence and charge state were listed in *Details in S2 Table.*

^3, 4^ The fold change in differentially expressed proteins between SBM and CSM_100._ Experiments were performed twice with 4 labeling iTRAQ regents.

^5,6^ The theoretical molecular weights and isoelectric points of the identified proteins were retrieved from the NCBInr protein database.

### Differential protein expression and validation by Western-blot

**Fig 3:**
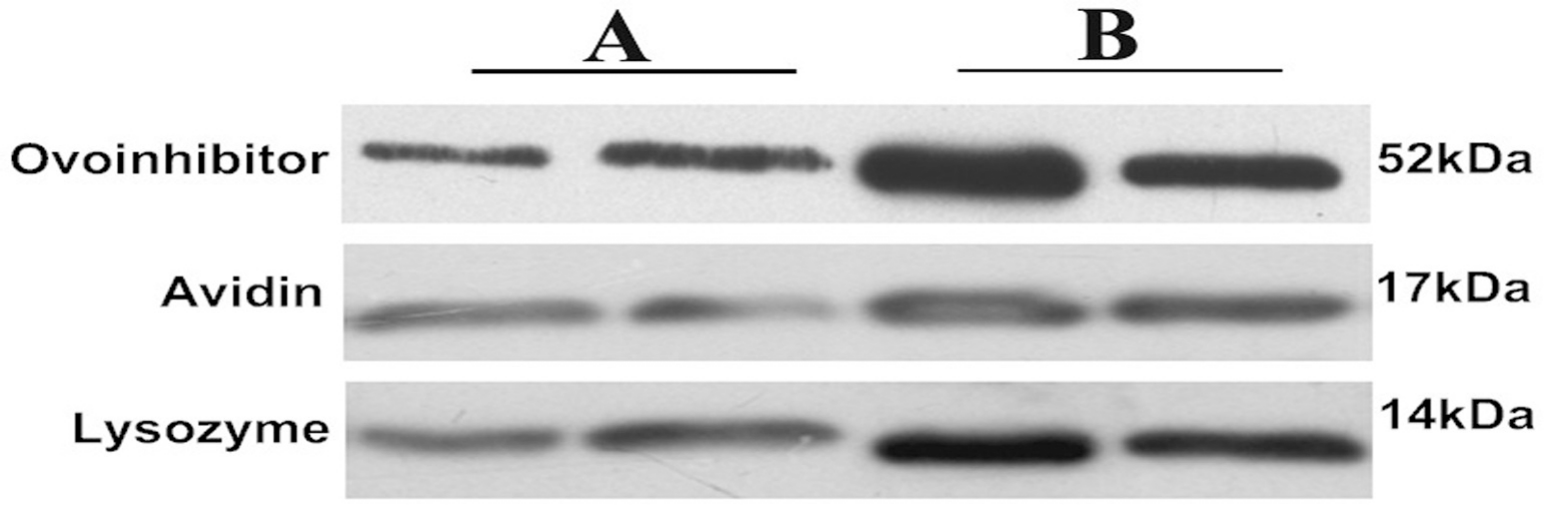
The protein expression of ovoinhibitor, avidin and lysozyme between laying hens fed diets formulated with soybean meal (SBM) and diets that replaced the 100% crude protein content of SBM with cottonseed meal (CSM_100_). A, B represent the CSM_100_ and SBM, respectively.

To confirm the iTRAQ results, 3 differentially expressed proteins; ovoinhibitor, lysozyme and avidin, were chosen for validation. Results show the same changes to the iTRAQ labeling (Fig 3).

## Discussion

This study revealed that feeding CSM could cause a decrease in laying performance and albumen quality. The link between egg weight and albumen weight is higher than that between egg weight and shell or yolk weight [13], and the reduction in egg weight was primarily associated with a reduction in albumen weight [14]. Our study confirmed that albumen weight was also significantly decreased in the CSM_100_ group compared with the control group.

HU is calculated from the height of the inner thick albumen and by egg weight [15]. HU and albumen height are important factors evaluating albumen quality [16]. HU was significantly decreased in the eggs of hens fed with 187 g/kg CSM [17]. The reduction of HU and albumen height in our study implied a decrease in the albumen quality of eggs in the CSM_100_ group. Results indicate individual egg white protein might be reduced in the CSM_100_ diet due to molecular mechanisms causing a loss in albumen quality.

Our study was the first to compare egg white proteins between SBM and CSM diets using iTRAQ, demonstrating that 15 proteins were down-regulated in the CSM_100_ diet compared with the SBM diet. Ovomucin is a highly glycosylated protein and accounts for approximately 3.5% of total egg white proteins. Ovomucin was undetectable in previous studies by 2-DE due to its large molecular weight. The glycoprotein ovomucin gives the egg white its thick viscous and gel-like properties. Ovomucin is the most important factor in determining the height of the inner thick albumen and HU [18]. The total amount of ovomucin isolated from the thick albumen of the eggs with high HU was much higher than the amount isolated from the low HU thick albumen [19]. This finding was consistent with our results showing that albumen height, HU and the intensity of ovomucin were significantly decreased in the CSM_100_ group. Reduction in viscosity values caused albumen thinning, directly affecting the shelf life of eggs [20]. The most accepted reason for egg white thinning is the degradation of ovomucin. A reduction of ovomucin in the CSM_100_ diet might provide an explanation for the poor albumen height and albumen quality in fresh or stored eggs. Similarly, eggs collected from hens fed with rapeseed meal-enriched diet were also characterized by poor storage stability [21]. Studies have shown ovomucin is partly present in egg white as a complex with lysozyme [22]. Lysozyme accounts for 3.5% of the total egg white protein and plays an important role in the protection of a developing embryo due to its effect on the biological function of bacteria and viruses. In this study, lysozyme was down-regulated in the CSM_100_ group compared to the SBM group. The incorporation of rapeseed meal lowered lysozyme activity [23]. Albumen viscosity might also depend on the ovomucin–lysozyme complex [22], which is stabilized by electrostatic interactions. Egg white thinning may result from interactions between ovomucin and lysozyme [24]. A reduction of both ovomucin and lysozyme in the CSM_100_ group supported the claim of poor albumen quality or stability with a CSM diet.

Clusterin is a type of chaperone found in biological fluids including semen, urine and human plasma. The unfolded or partly folded proteins cause the egg white to lose its viscous nature. Clusterin can lead to interaction and stabilization of unfolded or partly folded proteins, inhibiting the precipitation or aggregation of these proteins [25]. The increase in the level of clusterin may improve stabilization of partially unfolded proteins, thus improving protein stability and extending egg storage duration. Clusterin remained at a stable level at 4°C storage, but disappeared at high-temperature storage conditions, and albumen quality was decreased. The decrease of clusterin was suggested to be an effective biomarker for egg quality evaluation during the high-temperature storage [8]. Earlier studies reported on the decrease of albumen quality, such as development of albumen discoloration in stored eggs due to a CSM diet [4,18,26]. In our study, the reduced levels of clusterin in the CSM_100_ group may provide further evidence that albumen quality in stored eggs is reduced with laying hens fed a CSM diet.

Ovalbumin and ovotransferrin are glycoproteins, constituting 54% and 13% of the egg white protein, respectively. Ovotransferrin has defensive and protective activities against a wide range of bacteria. The ovalbumin is related to the viscosity, the gel-forming and foaming properties of egg white. Foam stability is important for the shelf-life of eggs [27]. The significant decrease of ovotransferrin and ovalbumin expression implied protective mechanisms in egg whites are reduced due to the biological function of these two proteins. Ovalbumin is likely involved in the changes that occur when albumen becomes less viscous [1]. Because ovalbumin is the major protein in egg whites, the presence of this protein can be used as a primary criterion as well as a sensitive test for the differentiation of egg white proteins [28]. Ovocalyxin-32 is a matrix protein found within the outer layers of the eggshell and in the cuticle. Ovocalyxin-32 had significant effects on albumen height and early egg weight [29]. It is difficult to hypothesize the role of the ovocalyxin-32 protein and albumen height as little is known about ovocalyxin-32 activity. Ovocalyxin-32 may have an influence on water and gas permeability through egg shell pores, ultimately influencing albumen viscosity. It could also be the effect of an unidentified, linked locus [29]. Chicken egg ovoinhibitor is a multidomain Kazal-type serine protease inhibitor and is highly expressed in the magnum, accouting for 1.5% of chicken egg white proteins. It has been reported that ovoinhibitor appeared to play a significant role in an antibacterial defense against *Bacillus subtilis* and *Staphylococcus aureus*[30]. The reduced abundance of ovoinhibitor may put adverse effect on the natural characterization of egg white protein in the CSM_100_ group.

The six decreased proteins: ovalbumen, ovotransferrin, ovomucin, lysozyme, ovoinhibitor and clusterin, account for almost 80% of egg white proteins, and highly relate to viscosity of the thick albumen [31]. Albumen height and HU measure the viscosity of the egg albumen[32]. Thus, these reduced high-abundance proteins might play an important role on the decrease of albumen height and albumen quality in the CSM_100_ group, indicating a high correlation between albumen height and their intensities in egg whites. It is interesting to note that four proteins related to immunity, including Ig mu chain C region, Ig J polypeptide, Ig heavy chain variable region and Ig alpha heavy chain, were identified to be decreased in the CSM_100_ group. These low-abundance proteins are involved with response to stimulus, immune system process, and might have a biological role in maintaining the integrity of the egg white, protecting early embryonic development [1].

Previous studies suggested that progesterone and estrogen are related to the synthesis of ovalbumin, ovotransferrin, ovomucoid, lysozyme and avidin [33,34]. The levels of progesterone and estrogen in plasma was not observed in the CSM_50_ group, but progesterone levels were significantly reduced in the CSM_100_ group. Progesterone plays an important role in the growth of epithelial cells and tubular gland cells of magnum [34,35]. In hens, the oviduct magnum is generally known as the formation site for egg protein synthesis. Results indicated less growth of epithelial cells and tubular gland cells in the CSM_100_ group compared with the control group. Thus, the histological findings were consistent with the plasma hormone data, demonstrating the CSM_100_ diet might cause a reduction of plasma progesterone levels in hens. This reduction further slowed tubular gland cells and epithelial cell growth in oviduct magnum during the feeding period. As well, a reduction of egg white protein synthesis was seen. With hundreds of proteins in egg whites, further investigation is needed, as few studies can be found between the specific cell type of magnum and synthesized proteins [35].

Waldroup (1981) recommended dietary FG level below 40 mg/kg in diets would be safe to hens. In this study, 20.16 mg/kg FG concentration in the CSM_100_ diet (Table 1) would not be sufficient to induce adverse effects [2,36]. The relationship between the adverse factors on quality induced by a CSM diet appears to have been oversimplified by earlier studies that ascribed all of the effects to FG in CSM [37]. The lowered levels of progesterone could be induced by some anti-nutritional factors in CSM such as malvalic and sterculic acid [35], or some gossypol-like components in the meal, and those chemical identities remain unknown [38]. Meanwhile, it might also be due to differences in the chemical composition between CSM and SBM diets. Different protein materials have a different composition of fiber types, which may contribute to different feeding results [39]. The feeding modification induced physiological reactions in the hens, expressed mainly by altered levels of biologically active substances, i.e., hormones in the blood. It was reported that albumen quality was decreased with the dietary addition of neem kernel meal [40]. Poor storage stability of eggs was also observed from hens fed with rapeseed meal-enriched diet [21]. In addition, CSM is high in arginine (Arg), and the increase in CSM in the CSM_100_ group greatly increased the Arg content, disturbing the Arg to lysine (Lys) ratio (S1 Table). An antagonism between Arg and Lys is well documented in poultry [41]. This antagonism might produce the hormone changes in hens.

Currently, there is little research focused on egg white proteins as a result of different types of proteins in hen diets. The molecular changes described in this study may explain the reason for the biochemical changes and suggest better selection criteria for chicken feed formula to produce more nutritive production levels.

## Conclusions

This study integrates traditional nutritional, histological and proteomic approaches to identify the effects of CSM on albumen quality. The CSM_100_ diet had an adverse effect on laying hen performance and egg albumen quality. This study identified 15 proteins involved with various biological functions, including antimicrobial activity, gelling characteristics, immune responses, inhibition of protein precipitation, protease inhibition, and metal ion binding or transport, that may play an important role in albumen quality reduction in the CSM_100_ group. The decrease in egg white proteins was induced by lower tubular gland and epithelial cell growth of the oviduct magnum when hens were fed a CSM_100_ diet. This study demonstrated at the molecular level that SBM should not be totally replaced with CSM in the diet of hens.

After the experiment, the remaining hens was fed using the control diet in the normal feeding conditions as described in Method part.

## Supporting information

S1 Table Total proteins identification in egg white of laying hens fed diets formulated with soybean meal (SBM) and diets replaced crude protein content of SBM with 100% cottonseed meal (CSM_100_). (DOCX)

S2 Table The sequence and charge state of significantly altered egg white proteins of laying hens fed diets formulated with soybean meal (SBM) and diets that replaced 100% crude protein content of SBM with cottonseed meal (CSM_100_). (DOCX)

S1 Fig The western-blot of ovoinhibitor, avidin, lysosome expressed in SBM and CSM_100_. (DOCX)

## Abbreviations

2-DE: two-dimensional electrophoresis
AA: amino acid
CSM: cottonseed meal
SBM: soybean meal
FG: free gossypol
HU: Haugh unit
iTRAQ: isobaric tags for relative and absolute quantification
MS: mass spectrometry
FCR: feed conversion ratio
