# Supplementary material for: Proteomic comparison by iTRAQ combined with mass spectrometry of egg white proteins in laying hens (Gallus gallus) fed with soybean meal and cottonseed meal
Source: PLoS One. 2017 Aug 15;12(8):e0182886. doi: 10.1371/journal.pone.0182886 (PMC5557595; doi:10.1371/journal.pone.0182886)
Supplement: S1 Table — (DOCX) [file pone.0182886.s001.docx]

**Supporting information**

**S1 Table** **Total proteins identification in e****gg whiteof laying hens fed diets formulated with soybean meal (SBM) and diets replaced crude protein content of SBM with 100% cottonseed meal (CSM_100_)**

| GI_Accession NO. | Description | Coverage | Proteins | Unique Peptides | Peptides | PSMs | SBM  /CSM_100_ | SBM  /CSM_100_ | average | cv | AAs | MW [kDa] | calc. pI |
| --- | --- | --- | --- | --- | --- | --- | --- | --- | --- | --- | --- | --- | --- |
| 63052 | unnamed protein product, partial | 57.42 | 2 | 1 | 8 | 422 | 1.008 | 1.037 | 1.02 | 0.02 | 155 | 17.46 | 5.52 |
| 113575 | Serum albumin | 21.14 | 2 | 11 | 11 | 15 | 1.163 | 1.148 | 1.15 | 0.01 | 615 | 69.87 | 5.74 |
| 118088 | Peptidyl-prolylcis-transisomerase B | 9.66 | 1 | 2 | 2 | 3 | 1.014 | 1.034 | 1.02 | 0.01 | 207 | 22.40 | 9.39 |
| 127513 | Ig mu chain C region | 8.74 | 1 | 4 | 4 | 7 | 1.348 | 1.337 | 1.34 | 0.01 | 446 | 48.14 | 6.42 |
| 129296 | Ovalbumin-related protein Y | 35.05 | 2 | 1 | 16 | 206 | 0.952 | 0.946 | 0.949 | 0.004 | 388 | 43.74 | 5.30 |
| 211055 | beta-actin | 6.98 | 19 | 1 | 1 | 1 | 0.621 | 0.609 | 0.61 | 0.01 | 86 | 9.34 | 5.12 |
| 211957 | Ig heavy chain V-region, partial | 14.95 | 6 | 1 | 1 | 1 | 1.033 | 1.098 | 1.07 | 0.04 | 107 | 11.13 | 9.25 |
| 212030 | Ig light chain, partial | 52.63 | 4 | 1 | 1 | 1 | 1.254 | 0.924 | 1.09 | 0.21 | 19 | 2.13 | 8.62 |
| 212164 | Ig light-chain VJ region, partial | 17.17 | 7 | 1 | 1 | 1 | 1.257 | 1.023 | 1.14 | 0.15 | 99 | 10.36 | 4.89 |
| 212748 | 190 kD tenascin precursor | 1.83 | 8 | 2 | 2 | 2 | 0.900 | 1.028 | 0.96 | 0.09 | 1532 | 168.52 | 5.24 |
| 223464 | ovomucoid | 76.34 | 4 | 3 | 15 | 355 | 1.062 | 1.100 | 1.08 | 0.31 | 186 | 20.18 | 4.91 |
| 227660 | secretory trypsin inhibitor | 13.43 | 1 | 1 | 1 | 1 | 0.856 | 0.790 | 0.82 | 0.06 | 67 | 7.38 | 5.06 |
| 229157 | lysozyme | 87.6 | 12 | 2 | 12 | 462 | 1.510 | 1.429 | 1.47 | 0.06 | 129 | 14.30 | 8.85 |
| 352173 | protein,riboflavin binding | 41.55 | 7 | 9 | 9 | 34 | 1.039 | 0.974 | 1.01 | 0.05 | 219 | 25.04 | 5.00 |
| 417552 | Pleiotrophin | 25 | 2 | 4 | 4 | 7 | 0.890 | 0.973 | 0.93 | 0.02 | 136 | 15.34 | 9.61 |
| 422699 | cell adhesion protein retina cognin | 1.85 | 5 | 1 | 1 | 1 | 1.169 | 0.988 | 1.08 | 0.12 | 378 | 42.44 | 4.69 |
| 576330 | Chain B, Crystal Structure Of Vitelline Membrane Outer Layer Protein I (Vmo-I): A Folding Motif With Homologous Greek Key Structures Related By An Internal Three-Fold Symmetry | 38.04 | 2 | 6 | 6 | 6 | 1.138 | 0.995 | 1.07 | 0.09 | 163 | 17.97 | 8.48 |
| 671865 | ovomacroglobulin, ovostatin | 23.45 | 5 | 31 | 31 | 72 | 1.114 | 1.177 | 1.14 | 0.02 | 1454 | 163.97 | 5.94 |
| 1480467 | mutant cysteine-rich FGF receptor | 3.34 | 2 | 4 | 4 | 4 | 1.062 | 1.067 | 1.06 | 0.00 | 1077 | 122.41 | 6.92 |
| 1536812 | Ig heavy chain variable region | 16.35 | 27 | 1 | 1 | 1 | 1.536 | 1.832 | 1.68 | 0.12 | 104 | 10.80 | 5.31 |
| 2498517 | Dickkopf-related protein 3 | 28.29 | 1 | 7 | 7 | 17 | 1.043 | 1.119 | 1.08 | 0.05 | 350 | 39.18 | 4.89 |
| 4033392 | 78 kDa glucose-regulated protein | 1.69 | 1 | 1 | 1 | 1 | 1.199 | 1.120 | 1.16 | 0.05 | 652 | 71.97 | 5.22 |
| 5689722 | aminopeptidase, partial | 2.86 | 4 | 1 | 1 | 1 | 0.838 | 0.946 | 0.89 | 0.09 | 559 | 63.50 | 5.10 |
| 5705960 | Ig alpha heavy chain | 6.15 | 2 | 3 | 3 | 7 | 2.207 | 2.227 | 2.22 | 0.01 | 585 | 61.47 | 4.72 |
| 6729945 | Ovotransferrin | 74.47 | 1 | 1 | 29 | 758 | 1.403 | 1.460 | 1.43 | 0.07 | 329 | 36.18 | 7.06 |
| 9887385 | CEPU-Se alpha 2 isoform | 12.78 | 4 | 4 | 4 | 4 | 1.151 | 1.057 | 1.10 | 0.06 | 313 | 34.46 | 6.30 |
| 44969651 | calreticulin, partial | 2.23 | 1 | 1 | 1 | 1 | 0.763 | 0.944 | 0.85 | 0.15 | 404 | 46.85 | 4.55 |
| 45382425 | vitamin D-binding protein precursor | 2.27 | 1 | 1 | 1 | 1 | 1.195 | 1.146 | 1.17 | 0.03 | 484 | 53.65 | 6.87 |
| 45382467 | clusterin precursor | 35.71 | 2 | 15 | 15 | 71 | 2.098 | 2.248 | 2.17 | 0.03 | 448 | 51.32 | 5.67 |
| 45382809 | ovomucin precursor | 37.43 | 2 | 64 | 64 | 273 | 1.598 | 1.682 | 1.64 | 0.01 | 2108 | 233.39 | 5.60 |
| 45382957 | prothrombin precursor | 1.81 | 1 | 1 | 1 | 1 | 1.451 | 1.435 | 1.44 | 0.01 | 607 | 69.07 | 5.66 |
| 45383039 | transient receptor potential cation channel subfamily V member 1 | 0.71 | 1 | 1 | 1 | 1 | 1.060 | 1.029 | 1.04 | 0.02 | 843 | 96.46 | 7.68 |
| 45383612 | prostaglandin-H2 D-isomerase precursor | 36.22 | 1 | 6 | 6 | 11 | 1.165 | 1.073 | 1.12 | 0.06 | 185 | 20.83 | 6.79 |
| 45384294 | transforming growth factor-beta-induced protein ig-h3 precursor | 4.26 | 1 | 3 | 3 | 3 | 0.881 | 0.974 | 0.93 | 0.07 | 680 | 74.37 | 7.59 |
| 46049078 | Ig J polypeptide | 8.86 | 1 | 1 | 1 | 1 | 1.737 | 1.285 | 1.51 | 0.21 | 158 | 17.93 | 5.35 |
| 50751384 | PREDICTED: di-N-acetylchitobiase | 3.3 | 1 | 1 | 1 | 1 | 0.926 | 1.526 | 1.23 | 0.35 | 364 | 40.66 | 5.14 |
| 58696426 | apolipoprotein D precursor | 35.98 | 1 | 7 | 7 | 14 | 1.103 | 1.113 | 1.11 | 0.01 | 189 | 21.69 | 5.80 |
| 71274079 | ovotransferrin BC type | 78.44 | 2 | 2 | 62 | 1785 | 0.844 | 0.904 | 0.87 | 0.03 | 705 | 77.76 | 7.24 |
| 82102958 | Sulfhydryl oxidase 1 | 9.42 | 1 | 6 | 6 | 8 | 0.963 | 0.895 | 0.93 | 0.05 | 743 | 83.03 | 7.40 |
| 83754919 | Chain A, Crystal Structure Of Aluminum-Bound Ovotransferrin At 2.15 Angstrom Resolution | 81.92 | 8 | 1 | 62 | 1810 | 0.881 | 0.954 | 0.92 | 0.06 | 686 | 75.81 | 7.01 |
| 93278504 | Chain I, The Plasmodium Falciparum Cysteine Protease Falcipain-2 | 31.53 | 6 | 3 | 3 | 13 | 1.175 | 1.081 | 1.13 | 0.06 | 111 | 12.57 | 7.77 |
| 113911467 | Gal 11 | 35.58 | 3 | 4 | 4 | 11 | 1.160 | 1.138 | 1.15 | 0.04 | 104 | 11.74 | 8.56 |
| 118100703 | PREDICTED: tumor necrosis factor receptor superfamily member 6B isoform X2 | 8.08 | 2 | 2 | 2 | 2 | 0.746 | 1.081 | 0.91 | 0.26 | 297 | 33.42 | 8.03 |
| 118103004 | PREDICTED: keratin, type I cytoskeletal 10-like isoform X1 | 3.43 | 11 | 2 | 2 | 2 | 0.909 | 0.852 | 0.88 | 0.05 | 466 | 51.13 | 4.84 |
| 118595739 | Trypsin inhibitor ClTI-1 | 50.91 | 3 | 2 | 2 | 3 | 1.019 | 1.196 | 1.11 | 0.11 | 55 | 6.03 | 6.35 |
| 123891643 | Olfactomedin-like protein 3 | 3.06 | 1 | 1 | 1 | 1 | 0.944 | 0.919 | 0.93 | 0.02 | 392 | 44.82 | 6.07 |
| 148238207 | protein FAM3C | 11.89 | 1 | 2 | 2 | 3 | 0.858 | 0.994 | 0.93 | 0.10 | 227 | 24.79 | 8.29 |
| 153792243 | polymeric immunoglobulin receptor precursor | 13.43 | 2 | 8 | 8 | 9 | 1.028 | 1.071 | 1.05 | 0.03 | 648 | 70.53 | 5.19 |
| 155624218 | alpha 1-acid glycoprotein | 25.62 | 2 | 6 | 6 | 118 | 0.959 | 0.924 | 0.94 | 0.03 | 203 | 22.31 | 5.25 |
| 157831653 | Chain A, Analysis Of The Stabilization Of Hen Lysozyme With The Helix Dipole And Charged Side Chains | 87.6 | 10 | 1 | 12 | 353 | 0.913 | 0.793 | 0.85 | 0.10 | 129 | 14.30 | 8.85 |
| 157831881 | Chain A, Three-Dimensional Structure Of The Platelet Integrin Recognition Segment Of The Fibrinogen Gamma Chain Obtained By Carrier Protein-Driven Crystallization | 82.64 | 51 | 1 | 13 | 401 | 1.137 | 1.004 | 1.07 | 0.09 | 144 | 15.86 | 9.01 |
| 157834076 | Chain A, Analysis Of The Stabilization Of Hen Lysozyme With The Helix Dipole And Charged Side Chains | 87.4 | 9 | 1 | 11 | 385 | 1.105 | 1.003 | 1.054 | 0.12 | 127 | 14.01 | 8.85 |
| 162952006 | ovomucoid precursor | 63.46 | 2 | 1 | 13 | 349 | 1.157 | 1.158 | 1.15 | 0.06 | 208 | 22.39 | 4.91 |
| 205361182 | angiopoietin-related protein 3 precursor | 11.42 | 1 | 4 | 4 | 5 | 1.021 | 1.049 | 1.03 | 0.02 | 464 | 53.45 | 5.25 |
| 253735708 | glutathione peroxidase 3 precursor | 14.61 | 1 | 3 | 3 | 5 | 0.877 | 0.910 | 0.89 | 0.03 | 219 | 24.63 | 8.60 |
| 257357678 | ovocalyxin-32 | 9.45 | 6 | 2 | 2 | 2 | 1.299 | 1.582 | 1.44 | 0.14 | 275 | 30.76 | 9.09 |
| 262367843 | Chain X, Structure Of N59d Hen Egg-white Lysozyme | 87.6 | 17 | 1 | 12 | 339 | 1.025 | 1.038 | 1.03 | 0.01 | 129 | 14.30 | 8.85 |
| 297787771 | Chain B, Crystal Structure Of Chicken Md-1 Complexed With Lipid Iva | 7.28 | 2 | 1 | 1 | 1 | 1.127 | 1.168 | 1.15 | 0.08 | 151 | 17.04 | 5.91 |
| 309318771 | ectonucleotidepyrophosphatase/phosphodiesterase 2 | 2.1 | 2 | 2 | 2 | 2 | 0.997 | 1.430 | 1.21 | 0.25 | 859 | 98.41 | 7.30 |
| 313667194 | interleukin 17F | 4.14 | 1 | 1 | 1 | 1 | 0.959 | 0.991 | 0.98 | 0.02 | 169 | 18.90 | 9.22 |
| 313747531 | leucine zipper protein 2 | 12.21 | 1 | 5 | 5 | 5 | 0.860 | 0.901 | 0.88 | 0.03 | 344 | 39.35 | 9.23 |
| 316995812 | Hep21 protein | 42.99 | 2 | 3 | 3 | 14 | 0.848 | 1.119 | 0.98 | 0.20 | 107 | 11.96 | 6.89 |
| 319443687 | Chain L, Crystal Structure Of The DegpDodecamer With A Model Substrate | 54.55 | 1 | 1 | 2 | 103 | 1.010 | 0.791 | 0.90 | 0.17 | 44 | 4.80 | 4.91 |
| 342165190 | Ovocleidin-116 | 5.92 | 2 | 2 | 2 | 3 | 1.117 | 1.112 | 1.11 | 0.00 | 743 | 76.82 | 6.92 |
| 363729530 | PREDICTED: hemopexin | 15.93 | 2 | 5 | 5 | 9 | 0.973 | 0.938 | 0.96 | 0.03 | 383 | 43.02 | 5.64 |
| 363733143 | carboxypeptidase E | 3.41 | 1 | 2 | 2 | 2 | 0.810 | 0.710 | 0.76 | 0.09 | 469 | 52.37 | 5.24 |
| 363734612 | PREDICTED: plasma protease C1 inhibitor isoform X1 | 2.78 | 1 | 1 | 1 | 1 | 1.780 | 0.995 | 1.39 | 0.40 | 503 | 54.45 | 6.10 |
| 363740281 | PREDICTED: complement component C8 gamma chain | 7.75 | 1 | 1 | 1 | 1 | 0.972 | 1.023 | 1.00 | 0.04 | 129 | 14.63 | 5.87 |
| 363744257 | PREDICTED: beta-hexosaminidase subunit beta | 1.97 | 1 | 1 | 1 | 1 | 0.988 | 1.046 | 1.02 | 0.04 | 558 | 62.97 | 6.39 |
| 365813052 | Chain B, The Siderocalin Ex-Fabp Functions Through Dual Ligand Specificities | 23.13 | 8 | 4 | 4 | 12 | 0.892 | 0.859 | 0.88 | 0.03 | 160 | 18.16 | 6.23 |
| 385145531 | ovoglobulinG2 type AB | 24.83 | 11 | 15 | 15 | 101 | 0.938 | 0.952 | 0.94 | 0.01 | 439 | 47.36 | 5.91 |
| 440923751 | Ovalbumin | 65.03 | 16 | 18 | 25 | 2202 | 1.456 | 1.480 | 1.468 | 0.01 | 386 | 42.88 | 5.29 |
| 510032768 | ovalbumin-related protein X | 31.59 | 3 | 5 | 13 | 134 | 1.017 | 1.225 | 1.12 | 0.13 | 402 | 45.40 | 6.74 |
| 513159520 | PREDICTED: metalloproteinase inhibitor 3-like | 6.36 | 2 | 1 | 1 | 1 | 0.960 | 1.196 | 1.08 | 0.16 | 173 | 20.20 | 9.25 |
| 513159546 | PREDICTED: BPI fold-containing family C protein isoform X2 | 3.41 | 3 | 2 | 2 | 3 | 1.478 | 0.872 | 1.17 | 0.36 | 469 | 52.28 | 8.35 |
| 513159615 | PREDICTED: BPI fold-containing family C protein-like | 4.76 | 1 | 3 | 3 | 3 | 0.960 | 0.809 | 0.88 | 0.12 | 525 | 58.29 | 7.66 |
| 513161014 | PREDICTED: alpha-2-macroglobulin-like 1 isoform X3 | 21.07 | 3 | 25 | 25 | 47 | 1.107 | 1.089 | 1.10 | 0.01 | 1310 | 146.62 | 8.24 |
| 513162189 | PREDICTED: LOW QUALITY PROTEIN: myelin protein zero-like 1 | 4.61 | 1 | 1 | 1 | 1 | 1.225 | 1.019 | 1.12 | 0.13 | 282 | 29.78 | 8.98 |
| 513179719 | PREDICTED: cell division cycle 5-like protein isoform X2 | 0.79 | 1 | 1 | 1 | 1 | 0.980 | 0.865 | 0.92 | 0.09 | 889 | 101.48 | 9.23 |
| 513186981 | PREDICTED: secretoglobin family 1C member 1-like, partial | 30 | 1 | 3 | 3 | 20 | 1.134 | 1.165 | 1.15 | 0.02 | 90 | 9.91 | 5.62 |
| 513188927 | PREDICTED: mucin-6 | 11.51 | 3 | 28 | 28 | 56 | 0.919 | 0.884 | 0.90 | 0.01 | 2677 | 291.12 | 7.56 |
| 513191195 | PREDICTED: beta-microseminoprotein-like | 30.19 | 1 | 3 | 3 | 16 | 0.944 | 0.916 | 0.93 | 0.12 | 106 | 12.13 | 8.94 |
| 513205480 | PREDICTED: protein FAM3D isoform X8 | 6.75 | 6 | 1 | 1 | 1 | 1.095 | 0.728 | 0.91 | 0.28 | 237 | 26.25 | 9.31 |
| 513205847 | PREDICTED: metabotropic glutamate receptor 7 isoform X3 | 0.65 | 2 | 1 | 1 | 3 | 0.984 | 0.769 | 0.88 | 0.17 | 929 | 103.18 | 7.90 |
| 513214451 | PREDICTED: NADPH:adrenodoxin oxidoreductase, mitochondrial isoform X8 | 1.74 | 8 | 1 | 1 | 1 | 0.765 | 1.094 | 0.93 | 0.25 | 461 | 50.11 | 9.07 |
| 513218610 | PREDICTED: uncharacterized protein LOC771972 isoformX1 | 6.12 | 2 | 1 | 1 | 2 | 0.938 | 0.986 | 0.96 | 0.09 | 245 | 27.06 | 8.37 |
| 513221142 | PREDICTED: platelet-activating factor acetylhydrolase 2 | 4.17 | 6 | 1 | 1 | 1 | 1.599 | 1.483 | 1.54 | 0.05 | 312 | 34.61 | 8.57 |
| 513226219 | PREDICTED: keratin, type I cytoskeletal 12 isoform X1 | 1.94 | 2 | 1 | 1 | 1 | 0.986 | 0.865 | 0.92 | 0.16 | 515 | 54.82 | 4.93 |
| 513226479 | PREDICTED: transmembrane protease serine 9 isoform X3 | 0.81 | 3 | 1 | 1 | 2 | 0.894 | 0.883 | 0.89 | 0.01 | 1114 | 119.97 | 6.68 |
| 513233729 | PREDICTED: deleted in malignant brain tumors 1 protein-like | 18.17 | 1 | 2 | 9 | 13 | 0.897 | 0.896 | 0.89 | 0.02 | 655 | 69.27 | 5.19 |
| 556503153 | EW135 | 15.1 | 5 | 5 | 12 | 17 | 1.058 | 1.081 | 1.07 | 0.02 | 987 | 104.27 | 4.92 |
| 558704994 | Avidin | 37.5 | 11 | 1 | 3 | 6 | 1.874 | 1.606 | 1.74 | 0.11 | 128 | 14.29 | 9.60 |
| 575403100 | Chain A, Nmr Structure Of Hen Egg Beta-defensinGallin (chicken Ovo-defensin) | 75.61 | 7 | 3 | 3 | 20 | 0.921 | 1.005 | 0.96 | 0.06 | 41 | 4.73 | 8.94 |
| 614458442 | Ig Y heavy chain constant region, partial | 3.24 | 3 | 1 | 1 | 1 | 0.972 | 1.018 | 0.99 | 0.03 | 401 | 42.61 | 6.52 |
| 746815865 | OvoDB1 | 14.52 | 1 | 1 | 1 | 1 | 1.250 | 1.386 | 1.32 | 0.07 | 62 | 7.10 | 9.57 |
