# Supplementary material for: Proteomic comparison by iTRAQ combined with mass spectrometry of egg white proteins in laying hens (Gallus gallus) fed with soybean meal and cottonseed meal
Source: PLoS One. 2017 Aug 15;12(8):e0182886. doi: 10.1371/journal.pone.0182886 (PMC5557595; doi:10.1371/journal.pone.0182886)
Supplement: S2 Table — (DOCX) [file pone.0182886.s002.docx]

**Supporting information**

**S2 Table The sequence and charge stateof significantly altered egg white proteins of laying hens fed diets formulated with soybean meal (SBM) and diets that replaced100% crude protein content of SBM with cottonseed meal (CSM_100_)**

| Accession | Description | Sequence | IonScore | Exp Value | Charge | MH+ [Da] | ΔM [ppm] |
| --- | --- | --- | --- | --- | --- | --- | --- |
| 127513 | Ig mu chain C region | mEcGLEPVVQQDIAIR | 65.44 | 7.84E-05 | 3 | 2002.030 | 4.017 |
|  |  | iQmNQSEGk | 40.11 | 0.020138 | 2 | 1338.695 | 2.144 |
|  |  | lETALGk | 38.35 | 0.025552 | 2 | 1019.635 | 0.969 |
|  |  | iQMNQSEGk | 31.19 | 0.184607 | 3 | 1322.705 | 5.721 |
|  |  | sATLTcR | 29.74 | 0.15798 | 2 | 952.499 | -1.118 |
|  |  | vISGPPYR | 19.85 | 2.23953 | 3 | 1032.595 | -0.872 |
| 211055 | beta-actin[Gallus gallus] | gILTLk | 21.84 | 0.375107 | 2 | 932.641 | 2.481 |
| 229157 | lysozyme | NTDGSTDYGILQINSR | 103.56 | 6.74E-09 | 2 | 1753.837 | 1.218 |
|  |  | nTDGSTDYGILQINSR | 103.52 | 1.15E-08 | 2 | 1897.936 | -0.553 |
|  |  | IVSDGDGMNAWVAWR | 100.87 | 9.01E-09 | 2 | 1676.791 | 3.585 |
|  |  | iVSDGDGmNAWVAWR | 89.77 | 1.55E-07 | 2 | 1836.883 | 0.585 |
|  |  | FESNFNTQATNR | 85.36 | 2.47E-07 | 2 | 1428.649 | -0.745 |
|  |  | IVSDGDGmNAWVAWR | 81.4 | 6.42E-07 | 2 | 1692.787 | 3.887 |
|  |  | kIVSDGDGmNAWVAWR | 80.71 | 2.61E-06 | 3 | 2109.081 | 0.787 |
|  |  | iVSDGDGMNAWVAWR | 79.94 | 1.8E-06 | 2 | 1820.891 | 2.088 |
|  |  | gYSLGNWVcAAk | 78.38 | 5.36E-06 | 2 | 1613.844 | 5.840 |
|  |  | nLcNIPcSALLSSDITASVNcAk | 76.71 | 6.17E-06 | 2 | 2796.393 | -0.081 |
|  |  | nTDGSTDyGILQINSR | 66.6 | 7.27E-05 | 2 | 2042.052 | 6.295 |
|  |  | fESNFNTQATNR | 65.1 | 5.04E-05 | 2 | 1572.762 | 5.991 |
|  |  | kIVSDGDGMNAWVAWR | 65.07 | 9.69E-05 | 3 | 2093.080 | -1.869 |
|  |  | gTDVQAWIR | 52.61 | 0.001509 | 2 | 1189.650 | 4.366 |
|  |  | cELAAAMk | 51.72 | 0.001754 | 2 | 1181.633 | 6.116 |
|  |  | HGLDNYR | 48.29 | 0.0011 | 2 | 874.419 | 3.282 |
|  |  | gYSLGNWVcAAK | 48.13 | 0.004343 | 2 | 1469.729 | -2.715 |
|  |  | rHGLDNYR | 47.13 | 0.005122 | 3 | 1174.618 | -1.229 |
|  |  | cELAAAmk | 44.9 | 0.006977 | 3 | 1197.627 | 4.842 |
|  |  | cELAAAmK | 41.03 | 0.01444 | 2 | 1053.526 | 6.252 |
|  |  | cELAAAMk | 37.73 | 0.039541 | 2 | 1037.530 | 5.801 |
|  |  | hGLDNYR | 36.8 | 0.043008 | 3 | 1018.520 | 1.684 |
|  |  | ckGTDVQAWIR | 27.7 | 0.525861 | 4 | 1621.877 | 2.643 |
|  |  | kIVSDGDGmNAWVAWR | 25.74 | 0.551773 | 3 | 1964.970 | -3.777 |
|  |  | cELAAAMkR | 17.02 | 5.86891 | 3 | 1337.737 | 7.525 |
|  |  | cELAAAMKR | 14.76 | 9.691656 | 3 | 1193.630 | 4.430 |
|  |  | wWcNDGR | 38.35 | 0.010045 | 2 | 1137.508 | 5.336 |
|  |  | hGLDNyR | 10.3 | 26.68174 | 3 | 1162.626 | 4.326 |
|  |  | cELAAAmK | 15.94 | 2.013269 | 2 | 909.417 | 0.411 |
|  |  | RHGLDNYR | 28.22 | 0.218759 | 3 | 1030.519 | 0.980 |
|  |  | gySLGNWVcAAk | 8.32 | 50.75797 | 3 | 1757.942 | 2.951 |
| 1536812 | Ig heavy chain variable region [Gallus gallus] | aLTQPASVSANPGETVk | 29.34 | 0.383871 | 2 | 1958.079 | -0.555 |
| 5705960 | Ig alpha heavy chain [Gallus gallus] | vTLLSDPTQEDPER | 49.67 | 0.002749 | 2 | 1743.901 | 7.231 |
|  |  | aQHAATGADVk | 43.91 | 0.009507 | 2 | 1356.757 | 7.330 |
|  |  | eQLQGNEFVcR | 37.58 | 0.021893 | 2 | 1523.738 | -0.749 |
|  |  | vTLLSDPTQEDPERR | 18.28 | 4.208913 | 2 | 1900.000 | 5.778 |
|  |  | dGDFYSLYSk | 10.6 | 15.15477 | 3 | 1482.744 | 5.688 |
| 6729945 | Ovotransferrin | aIANNEADAISLDGGQVFEAGLAPYk | 124.59 | 1.18E-10 | 3 | 2922.504 | -1.512 |
|  |  | gAIEWEGIESGSVEQAVAk | 109.75 | 3.34E-09 | 2 | 2248.184 | 6.155 |
|  |  | nAPYSGYSGAFHcLk | 99.26 | 2.12E-08 | 2 | 1959.960 | -1.308 |
|  |  | gTEFTVNDLQGk | 80.97 | 2.17E-06 | 2 | 1596.857 | 6.121 |
|  |  | NAPYSGYSGAFHcLk | 72.85 | 5.68E-06 | 3 | 1815.862 | 0.840 |
|  |  | ISLTcVQk | 72.14 | 1.29E-05 | 2 | 1092.627 | 5.838 |
|  |  | sDFHLFGPPGk | 71.79 | 1.87E-05 | 2 | 1489.812 | 5.018 |
|  |  | kGTEFTVNDLQGk | 71.36 | 1.97E-05 | 2 | 1869.045 | 0.521 |
|  |  | hTTVNENAPDQkDEYELLcLDGSR | 71.04 | 1.68E-05 | 2 | 3092.496 | 4.037 |
|  |  | fFSAScVPGATIEQk | 69.39 | 3.21E-05 | 2 | 1930.002 | 1.653 |
|  |  | GAIEWEGIESGSVEQAVAk | 68.92 | 3.7E-05 | 2 | 2104.080 | 5.770 |
|  |  | AQSDFGVDTk | 65.61 | 3.5E-05 | 2 | 1211.607 | 3.703 |
|  |  | KGTEFTVNDLQGK | 65.13 | 6.43E-05 | 2 | 1436.741 | 1.807 |
|  |  | aTYLDcIk | 65.03 | 6.51E-05 | 2 | 1271.699 | 6.177 |
|  |  | iSLTcVQk | 64.31 | 7.44E-05 | 2 | 1236.728 | 4.160 |
|  |  | vEDIWSFLSk | 64.22 | 0.000102 | 2 | 1511.844 | 5.920 |
|  |  | dEYELLcLDGSR | 63.38 | 4.21E-05 | 2 | 1613.767 | 4.163 |
|  |  | dDNKVEDIWSFLSk | 62.29 | 0.00018 | 2 | 1984.030 | 1.667 |
|  |  | vAAHAVVAR | 61.58 | 8.88E-05 | 3 | 1037.632 | -1.327 |
|  |  | aQSDFGVDTk | 61.04 | 0.00017 | 2 | 1355.713 | 6.002 |
|  |  | dDNkVEDIWSFLSk | 60.82 | 0.000262 | 2 | 2128.143 | 6.883 |
|  |  | FFSAScVPGATIEQK | 59.13 | 0.000141 | 2 | 1641.796 | 1.289 |
|  |  | kGTEFTVNDLQGk | 56.03 | 0.000766 | 2 | 1724.950 | 4.817 |
|  |  | FFSAScVPGATIEQk | 56.03 | 0.000489 | 2 | 1785.896 | 0.017 |
|  |  | dSAIMLk | 55.2 | 0.000683 | 2 | 1065.627 | 5.225 |
|  |  | GTEFTVNDLQGk | 52.27 | 0.001285 | 2 | 1452.755 | 6.820 |
|  |  | sDFHLFGPPGkk | 47.76 | 0.004366 | 2 | 1762.011 | 5.414 |
|  |  | aIANNEADAISLDGGQVFEAGLAPyk | 45.78 | 0.008724 | 3 | 3066.606 | -1.584 |
|  |  | SDFHLFGPPGk | 45.31 | 0.006036 | 2 | 1345.710 | 5.836 |
|  |  | ATYLDcIk | 42.61 | 0.007081 | 2 | 1127.589 | 0.048 |
|  |  | tScHTGLGR | 41.84 | 0.006815 | 2 | 1132.568 | 2.911 |
|  |  | gDVAFVk | 40.34 | 0.016732 | 2 | 1023.613 | 4.986 |
|  |  | dGkGDVAFVk | 39.87 | 0.021381 | 2 | 1467.859 | 4.163 |
|  |  | HTTVNENAPDQkDEYELLcLDGSR | 39.2 | 0.022025 | 3 | 2948.408 | 8.913 |
|  |  | TScHTGLGR | 36.6 | 0.006443 | 3 | 988.462 | -1.421 |
|  |  | VAAHAVVAR | 33.83 | 0.07245 | 2 | 893.534 | 2.176 |
|  |  | HTTVNENAPDQKDEYELLcLDGSR | 32.92 | 0.048524 | 4 | 2804.294 | 5.366 |
|  |  | wcTVSSPEEk | 31.34 | 0.138639 | 2 | 1510.756 | 7.192 |
|  |  | dEyELLcLDGSR | 31.15 | 0.124926 | 3 | 1757.872 | 5.648 |
|  |  | qPVDNYKTcNWAR | 31.13 | 0.114518 | 3 | 1795.876 | 4.930 |
|  |  | aTyLDcIk | 30.39 | 0.21276 | 2 | 1415.801 | 5.884 |
|  |  | NAPYSGYSGAFHcLK | 29.05 | 0.059861 | 3 | 1671.761 | 1.394 |
|  |  | nAPYSGYSGAFHcLKDGk | 26.49 | 0.446533 | 4 | 2260.102 | -1.883 |
|  |  | HTTVNENAPDQk | 26.43 | 0.390065 | 3 | 1497.748 | 4.655 |
|  |  | aQSDFGVDTkSDFHLFGPPGk | 23.93 | 1.355127 | 4 | 2682.406 | 6.532 |
|  |  | hTTVNENAPDQkDEyELLcLDGSR | 22.37 | 1.493188 | 4 | 3236.589 | 1.243 |
|  |  | SDFHLFGPPGkk | 20.07 | 2.82362 | 4 | 1617.899 | -0.300 |
|  |  | gDVAFVkHTTVNENAPDQkDEYELLcLDGSR | 12.87 | 14.53196 | 4 | 3952.976 | 1.196 |
|  |  | AIANNEADAISLDGGQVFEAGLAPYk | 4.18 | 118.5173 | 3 | 2778.398 | -3.014 |
|  |  | GDVAFVk | 38.92 | 0.025486 | 2 | 879.506 | 0.334 |
|  |  | nAPYSGySGAFHcLk | 7.05 | 51.36189 | 4 | 2104.062 | -1.411 |
|  |  | dSAImLk | 28.66 | 0.314834 | 3 | 1081.619 | 1.880 |
|  |  | qPVDNYk | 21.02 | 1.801561 | 3 | 1151.631 | 0.565 |
|  |  | dGKGDVAFVk | 12.88 | 12.91678 | 3 | 1323.749 | -1.633 |
|  |  | dPVLKDLLFk | 25.71 | 0.263164 | 3 | 1475.915 | 5.066 |
|  |  | DSAIMLK | 40.04 | 0.021486 | 2 | 777.418 | 0.991 |
|  |  | kDPVLk | 28.07 | 0.102541 | 3 | 1131.746 | 0.115 |
|  |  | cNNLRDLTQQER | 14.34 | 8.108041 | 3 | 1690.851 | 6.011 |
|  |  | kcNNLRDLTQQER | 21.32 | 2.395237 | 3 | 1963.045 | 3.524 |
|  |  | dLTQQER | 19.19 | 1.82804 | 2 | 1033.540 | 0.819 |
| 45382467 | clusterin precursor [Gallus gallus] | mLDGGHGAWDHLLGGFESESR | 85.36 | 3.64E-07 | 2 | 2431.142 | 8.148 |
|  |  | eHQAMLHTLEETk | 71.61 | 1.95E-05 | 3 | 1854.963 | 0.557 |
|  |  | yIDTEVENAINGVk | 63.95 | 0.000118 | 3 | 1853.003 | 7.355 |
|  |  | mLDGGHGAWDHLLGGFESESR | 63.75 | 5.22E-05 | 3 | 2415.135 | 3.463 |
|  |  | eHQAMLHTLEETkR | 60.67 | 0.000296 | 2 | 2011.080 | 8.207 |
|  |  | eLHPFLQHPVHGFHR | 59.85 | 0.000356 | 2 | 1995.067 | 4.599 |
|  |  | eHQAmLHTLEETk | 58.15 | 0.000385 | 3 | 1870.955 | -1.264 |
|  |  | eILAVDcSQTDPVQSQLR | 51.09 | 0.002203 | 2 | 2203.127 | 5.740 |
|  |  | qLSAAGSk | 49.54 | 0.002469 | 2 | 1049.624 | 4.723 |
|  |  | icHSGSGLVGR | 45.45 | 0.005086 | 3 | 1286.678 | 2.193 |
|  |  | IDALLDR | 42.93 | 0.005473 | 2 | 815.465 | 3.835 |
|  |  | eHQAmLHTLEETkR | 41.11 | 0.026963 | 3 | 2027.068 | 4.863 |
|  |  | QLSAAGSk | 36.98 | 0.056556 | 2 | 905.520 | 3.399 |
|  |  | iDALLDR | 35.56 | 0.048742 | 2 | 959.565 | 0.575 |
|  |  | sSPFSIWVNGER | 35.45 | 0.066514 | 2 | 1522.783 | 3.869 |
|  |  | eAFVPPVQR | 32.62 | 0.107625 | 3 | 1186.670 | -0.270 |
|  |  | EAFVPPVQR | 31.28 | 0.105342 | 2 | 1042.574 | 5.717 |
|  |  | eQFEDALR | 20.75 | 1.117793 | 2 | 1151.581 | 0.090 |
|  |  | rkEEAVk | 11.71 | 9.099383 | 4 | 1291.803 | -2.192 |
|  |  | nSAGcLR | 28.61 | 0.201279 | 2 | 921.473 | 4.557 |
|  |  | qLEELLNR | 23.21 | 1.02621 | 3 | 1158.661 | 1.055 |
|  |  | iDALLDREQR | 14.92 | 8.336126 | 3 | 1372.766 | -0.719 |
|  |  | TPPFGGFR | 31.12 | 0.107828 | 2 | 878.453 | 0.615 |
|  |  | lFEMTQR | 18.7 | 2.157666 | 2 | 1068.564 | 1.065 |
| 45382809 | ovomucin precursor [Gallus gallus] | TATGAVEDSAAAFGNSWk | 112.38 | 1.08E-09 | 2 | 1926.942 | 5.306 |
|  |  | aPcTDGcFcPPGTILDDLGGk | 111.16 | 8.52E-10 | 2 | 2539.184 | -1.150 |
|  |  | tSGLcGNFNNIQTDDFR | 93.43 | 3.6E-08 | 2 | 2102.967 | -0.556 |
|  |  | gQSVEMSIQEFGNSWk | 91.31 | 1.57E-07 | 2 | 2115.057 | 6.951 |
|  |  | qGIcDPSEEcPETMVYNYSVk | 89.11 | 1.01E-07 | 2 | 2794.287 | 9.252 |
|  |  | sEVALcSVLSTYSR | 87.74 | 3.73E-07 | 2 | 1715.886 | 5.927 |
|  |  | vVPPQPYYEAcVASR | 84.23 | 1.01E-06 | 2 | 1879.959 | 5.351 |
|  |  | iQEIATDPGAEk | 83.8 | 1.02E-06 | 2 | 1559.864 | 7.675 |
|  |  | tATGAVEDSAAAFGNSWk | 83.06 | 1.25E-06 | 2 | 2071.036 | 1.158 |
|  |  | TSGLcGNFNNIQTDDFR | 82.3 | 2.26E-07 | 2 | 1958.878 | 6.139 |
|  |  | nTDDTFVVIGEIIQcGTSk | 79.94 | 3.07E-06 | 2 | 2385.226 | 1.909 |
|  |  | vcLPFEESNcVPGTVDVTSDGcck | 77.61 | 1.21E-06 | 2 | 3018.373 | 5.617 |
|  |  | qGIcDPSEEcPETmVYNYSVk | 74.39 | 2.34E-06 | 2 | 2810.271 | 5.101 |
|  |  | vDcNTcTcNk | 74.06 | 1.16E-06 | 2 | 1559.694 | 5.624 |
|  |  | ycNPGISEPVk | 71.89 | 1.56E-05 | 2 | 1551.809 | 0.904 |
|  |  | IcGLcGNYDGNk | 71.72 | 3.2E-06 | 2 | 1514.690 | 3.143 |
|  |  | nDLILDGYk | 66.09 | 5.66E-05 | 2 | 1338.757 | 4.386 |
|  |  | iVIQNNAcGk | 65.96 | 6.08E-05 | 2 | 1404.789 | 1.547 |
|  |  | lSSITcPPQQLk | 65.35 | 8.1E-05 | 2 | 1659.933 | -0.950 |
|  |  | vELVcSEHk | 64.87 | 7.91E-05 | 2 | 1388.752 | 5.036 |
|  |  | dcAAAGMTLk | 62.58 | 0.000105 | 2 | 1325.686 | 5.058 |
|  |  | aPcTDGcFcPPGTILDDLGGkk | 60.19 | 0.00026 | 3 | 2811.399 | 5.227 |
|  |  | dScPcMFQGk | 59.35 | 4.84E-05 | 2 | 1517.688 | 5.816 |
|  |  | iENYQHcEPSELck | 58.64 | 0.000154 | 2 | 2094.993 | 4.889 |
|  |  | eVIVDTLLSR | 58.06 | 0.000308 | 3 | 1288.756 | -2.836 |
|  |  | iTLIFESSEIR | 54.77 | 0.000832 | 2 | 1451.821 | -0.830 |
|  |  | fSYVHVDEcGcVETk | 52.04 | 0.000636 | 3 | 2117.990 | 1.401 |
|  |  | vQIAPmEGcGcPEGTYLNDEEEcVTPDDcPcYYk | 51.6 | 5.5E-05 | 4 | 4360.824 | 4.582 |
|  |  | fSyVHVDEcGcVETk | 50.91 | 0.001519 | 3 | 2262.100 | 4.517 |
|  |  | dcAAAGmTLk | 50.74 | 0.001354 | 2 | 1341.682 | 5.422 |
|  |  | IVQPGNSFQEDk | 48.88 | 0.002758 | 2 | 1505.779 | 4.999 |
|  |  | nGEVTDSFk | 48.17 | 0.002967 | 3 | 1284.674 | 4.740 |
|  |  | lDcIGETVLVk | 47.81 | 0.00427 | 2 | 1534.873 | -1.464 |
|  |  | gVcVSEGVEFkPGAVVPk | 47.71 | 0.003735 | 2 | 2291.295 | 6.882 |
|  |  | vcGLcGDFDGR | 46.62 | 0.000871 | 2 | 1399.629 | 5.201 |
|  |  | iIWNLTEcHR | 46.62 | 0.004809 | 2 | 1485.780 | 3.443 |
|  |  | IVIQNNAcGk | 46.38 | 0.005182 | 2 | 1260.693 | 5.994 |
|  |  | tTVVVHVTPSFQGk | 45.11 | 0.006882 | 3 | 1788.034 | 4.582 |
|  |  | eQYIVHk | 44.96 | 0.007843 | 2 | 1204.699 | 4.830 |
|  |  | sSVFEAcHSk | 44.89 | 0.005475 | 2 | 1439.730 | 7.216 |
|  |  | iVQPGNSFQEDk | 44.85 | 0.00956 | 2 | 1649.882 | 5.147 |
|  |  | cIccHEk | 44.72 | 0.001987 | 2 | 1294.601 | 4.659 |
|  |  | tcIDLPHk | 44.04 | 0.008207 | 2 | 1271.705 | 2.233 |
|  |  | cQTTcQQGFR | 43.32 | 0.000736 | 2 | 1285.546 | 4.026 |
|  |  | dQVyKPcGEAk | 42.86 | 0.015652 | 4 | 1726.918 | 1.236 |
|  |  | gTNLVLFNDkk | 42.66 | 0.008848 | 2 | 1680.996 | -2.982 |
|  |  | tQDmHcYVTEcVSGcmcPDGLVLDGSGGcIPk | 42.6 | 0.001085 | 4 | 3923.673 | -0.469 |
|  |  | LDcIGETVLVk | 42.24 | 0.016054 | 2 | 1390.780 | 4.625 |
|  |  | YcNPGISEPVk | 41.82 | 0.012183 | 2 | 1407.711 | 3.520 |
|  |  | iQcVPVk | 41.77 | 0.011725 | 2 | 1131.684 | 3.780 |
|  |  | tPAIcPVFcDYYNPPDk | 39.34 | 0.020128 | 3 | 2345.130 | 4.918 |
|  |  | QGIcDPSEEcPETMVYNYSVk | 39.16 | 0.002221 | 3 | 2650.126 | -12.378 |
|  |  | eLcYQEcPSNmEYMEcGNScADTcADPER | 38.95 | 0.00028 | 3 | 3736.399 | 3.751 |
|  |  | cQTTcQQGFR | 38.71 | 0.006292 | 2 | 1429.653 | 6.941 |
|  |  | kNDLILDGYk | 38.08 | 0.026934 | 2 | 1610.947 | -0.605 |
|  |  | cGNILYSLEGcYPEcSPDkPYFDEER | 37.47 | 0.010994 | 4 | 3486.545 | -2.319 |
|  |  | icGLcGNYDGNk | 37.35 | 0.022577 | 3 | 1658.800 | 8.006 |
|  |  | dQcPcVHGGHFYkPGETIR | 36.02 | 0.046794 | 3 | 2546.233 | 2.481 |
|  |  | cMYDTcNAEk | 34.05 | 0.010685 | 3 | 1579.684 | 2.827 |
|  |  | icGLcGNYDGNkk | 34.02 | 0.100357 | 2 | 1930.996 | 6.372 |
|  |  | sLSIcSLk | 33.89 | 0.081174 | 2 | 1195.698 | 2.006 |
|  |  | gQSVEmSIQEFGNSWk | 33.69 | 0.087757 | 2 | 2131.045 | 3.900 |
|  |  | kVVPDISk | 33.1 | 0.02613 | 2 | 1317.859 | 9.522 |
|  |  | qFGNFHk | 32.35 | 0.130944 | 2 | 1165.642 | 5.446 |
|  |  | ycNPGISEPVk | 31.5 | 0.18265 | 3 | 1695.905 | -2.715 |
|  |  | LSSITcPPQQLk | 31.23 | 0.187736 | 3 | 1515.843 | 6.891 |
|  |  | eMALDWk | 31.08 | 0.155264 | 3 | 1180.634 | 5.513 |
|  |  | VVPPQPYYEAcVASR | 30.78 | 0.143055 | 2 | 1735.851 | 2.427 |
|  |  | ecVSLPDcTScNPEEk | 28.99 | 0.089842 | 3 | 2212.999 | 10.206 |
|  |  | iLSSAGVQIR | 28.87 | 0.182513 | 3 | 1187.730 | 5.823 |
|  |  | nVLVTLGR | 27.72 | 0.180962 | 2 | 1015.639 | 0.746 |
|  |  | iENyQHcEPSELck | 27.59 | 0.322147 | 3 | 2239.095 | 4.781 |
|  |  | sAAPVPVPFcEGTcSTySVYSFENNEMEHk | 26.6 | 0.311865 | 4 | 3869.807 | 6.411 |
|  |  | vVPPQPYyEAcVASR | 26.3 | 0.692837 | 3 | 2024.052 | 0.404 |
|  |  | kSHVEk | 26.11 | 0.299398 | 3 | 1159.717 | 0.961 |
|  |  | gTcTVYGNGHYMSFDGEk | 26 | 0.122706 | 3 | 2311.042 | 2.508 |
|  |  | dQVYKPcGEAk | 25.97 | 0.65003 | 3 | 1582.825 | 7.304 |
|  |  | rEcVSLPDcTScNPEEk | 23.56 | 0.318081 | 3 | 2369.083 | 2.249 |
|  |  | cmYDTcNAEk | 22.44 | 0.099494 | 3 | 1595.677 | 1.472 |
|  |  | dScPcmFQGk | 20.88 | 0.189039 | 3 | 1533.677 | 2.038 |
|  |  | sEcTTWGNFHFHTFDHVk | 19.74 | 1.601568 | 4 | 2538.191 | 1.667 |
|  |  | cPDVRPDDHTGR | 16.84 | 1.819654 | 4 | 1568.741 | 3.469 |
|  |  | cmyDTcNAEk | 16.13 | 1.143333 | 3 | 1739.781 | 2.781 |
|  |  | qGIcDPSEEcPETmVyNYSVk | 15.66 | 2.534438 | 3 | 2954.359 | 0.340 |
|  |  | sPPINcTk | 15.17 | 7.670632 | 3 | 1204.662 | 1.658 |
|  |  | ycNPGISEPVKIENyQHcEPSELck | 14.43 | 7.319746 | 5 | 3483.663 | -2.193 |
|  |  | eQyIVHk | 8.97 | 25.66995 | 3 | 1348.795 | -0.094 |
|  |  | qWNcTDNPck | 12.7 | 3.010063 | 3 | 1610.731 | 1.212 |
|  |  | vVPDISk | 38.86 | 0.019756 | 2 | 1045.652 | 2.322 |
|  |  | lAFYIk | 31.35 | 0.065954 | 2 | 1042.654 | -0.195 |
|  |  | emALDWk | 26.29 | 0.384635 | 3 | 1196.631 | 7.266 |
|  |  | hcSIIk | 13.74 | 10.39553 | 3 | 1045.611 | 4.603 |
|  |  | dcVLSk | 33.81 | 0.08443 | 2 | 1009.560 | 1.398 |
|  |  | qGIcDPSEEcPETMVyNYSVk | 9.26 | 13.69563 | 3 | 2938.375 | 3.991 |
|  |  | icGLcGNyDGNk | 9.73 | 21.37863 | 3 | 1802.900 | 6.208 |
|  |  | tcIDLPHK | 9.07 | 17.93158 | 3 | 1127.600 | -0.405 |
|  |  | kNDLILDGYk | 19.59 | 2.704653 | 2 | 1466.841 | -3.569 |
|  |  | vDcNTcTcNkR | 11.72 | 4.700742 | 3 | 1715.793 | 3.975 |
|  |  | sEVALcSVLSTySR | 4.28 | 101.2068 | 3 | 1859.985 | 4.174 |
|  |  | vQIAPmEGcGcPEGTyLNDEEEcVTPDDcPcYYk | 8.86 | 1.553703 | 4 | 4504.911 | 1.181 |
|  |  | nDLILDGyk | 5.33 | 65.68132 | 3 | 1482.852 | -0.338 |
|  |  | fGNcPk | 29.26 | 0.167431 | 2 | 1010.533 | 0.250 |
| 45382957 | prothrombin precursor [Gallus gallus] | sGIEcQVWTSk | 28.22 | 0.36942 | 2 | 1582.821 | 4.304 |
|  |  | vkPSTTGQPcESEk | 2.08 | 196.5796 | 3 | 1980.054 | 5.329 |
| 46049078 | Ig J polypeptide, [Gallus gallus] | hIQAALTPTScYAE | 34.28 | 0.058656 | 2 | 1705.835 | 1.024 |
|  |  | fVPSkDNPEEEVLER | 12.92 | 17.15807 | 3 | 2076.092 | 3.117 |
| 257357678 | ovocalyxin-32  [Gallus gallus] | qSTEHTGYLLAQVSSVk | 28.84 | 0.420848 | 4 | 2136.151 | -1.598 |
|  |  | fYEYLQHQk | 34.76 | 0.08517 | 2 | 1543.824 | 6.251 |
| 363733143 | carboxypeptidase E [Gallus gallus] | aASQPGELk | 26.91 | 0.448557 | 2 | 1188.692 | 8.085 |
|  |  | iVYVNEk | 28.51 | 0.221047 | 2 | 1152.689 | 1.585 |
|  |  | yVGNmHGNEAVGR | 17.8 | 1.661246 | 3 | 1563.742 | -1.940 |
| 513221142 | platelet-activating factor acetylhydrolase2[Gallus gallus] | mGGEQSLALPPEk | 21.04 | 2.38829 | 3 | 1660.893 | 6.742 |
| 558704994 | Avidin | fSESTTVFTGQcFIDR | 61.2 | 0.000118 | 2 | 2038.978 | 5.799 |
|  |  | eSPLHGTQNTINk | 49.23 | 0.003666 | 3 | 1726.931 | -1.170 |
|  |  | gEFTGTYTTAVTATSNEIk | 43.94 | 0.013106 | 3 | 2279.171 | 2.918 |
|  |  | gEFTGTyTTAVTATSNEIk | 16.2 | 8.646593 | 4 | 2423.262 | -1.775 |
|  |  | sSVNDIGDDWk | 18.94 | 2.113783 | 2 | 1523.762 | 2.594 |
| 746815865 | OvoDB1 [Gallus gallus] | gYcAPTcNk | 43.68 | 0.003358 | 2 | 1358.650 | 4.631 |
| 762068625 | ovoinhibitor precursor [Gallus gallus] | iLSPVcGTDGFTYDNEcGIcAHNAEQR | 107.52 | 9.6E-10 | 3 | 3228.441 | 4.308 |
|  |  | iLSPVcGTDGFTyDNEcGIcAHNAEQR | 82.34 | 4.03E-07 | 4 | 3372.529 | 0.153 |
|  |  | qEIPEIDcDQYPTR | 79.42 | 1.2E-06 | 2 | 1907.898 | 3.075 |
|  |  | lEIGSVDcSk | 65.22 | 7.32E-05 | 2 | 1395.748 | 6.175 |
|  |  | nLkPVcGTDGSTYSNEcGIcLYNR | 64.05 | 5.17E-05 | 4 | 3066.446 | 4.552 |
|  |  | cFFcNAYVQSNR | 57.41 | 0.000105 | 2 | 1709.774 | 5.611 |
|  |  | vSPIcTmEYVPHcGSDGVTYSNR | 57.05 | 0.000144 | 2 | 2789.267 | 7.748 |
|  |  | yPSTVSk | 56.15 | 0.000349 | 2 | 1069.620 | 6.295 |
|  |  | dGNTMVAcPR | 55.31 | 0.000198 | 2 | 1264.591 | 0.971 |
|  |  | dGTSWVAcPR | 50.2 | 0.000749 | 2 | 1292.617 | -0.629 |
|  |  | iLLPVcGTDGFTYDNEcGIcAHNAQHGTEVk | 48.6 | 0.001753 | 5 | 3764.772 | 0.296 |
|  |  | dGNTmVAcPR | 47 | 0.000987 | 3 | 1280.586 | 1.273 |
|  |  | nLKPVcGTDGSTYSNEcGIcLYNR | 46.09 | 0.001984 | 3 | 2922.340 | 3.240 |
|  |  | vSPIcTMEYVPHcGSDGVTYSNR | 40.72 | 0.006833 | 4 | 2773.270 | 7.226 |
|  |  | nLkPVcGTDGSTySNEcGIcLYNR | 40 | 0.020835 | 3 | 3210.554 | 6.154 |
|  |  | tLNLVSmAAc | 38.25 | 0.017828 | 2 | 1239.616 | -2.643 |
|  |  | LEIGSVDcSk | 38.05 | 0.028358 | 2 | 1251.649 | 9.334 |
|  |  | hVTIDcSPYLQVVR | 37.33 | 0.049459 | 4 | 1830.967 | 0.686 |
|  |  | ILSPVcGTDGFTYDNEcGIcAHNAEQR | 33.66 | 0.014853 | 3 | 3084.344 | 6.374 |
|  |  | DGNTmVAcPR | 33.02 | 0.007184 | 2 | 1136.483 | 0.517 |
|  |  | cRQEIPEIDcDQYPTR | 30.04 | 0.095566 | 3 | 2224.033 | 3.648 |
|  |  | eHGANVEk | 27.48 | 0.315762 | 2 | 1171.638 | 6.391 |
|  |  | hVTIDcSPyLQVVR | 27.13 | 0.55372 | 2 | 1975.083 | 8.060 |
|  |  | lHDGEckLEIGSVDcSk | 23.68 | 1.300645 | 4 | 2379.217 | 6.939 |
|  |  | vSPIcTmEyVPHcGSDGVTYSNR | 20.69 | 0.765231 | 4 | 2933.347 | 0.072 |
|  |  | vSPIcTMEYVPHcGSDGVTySNR | 13.86 | 4.11972 | 4 | 2917.354 | 0.505 |
|  |  | qEIPEIDcDQyPTR | 13.3 | 7.62876 | 3 | 2051.988 | -3.221 |
|  |  | vSPIcTmEyVPHcGSDGVTySNR | 4.7 | 46.57413 | 4 | 3077.450 | 0.223 |
|  |  | iLLPVcGTDGFTyDNEcGIcAHNAQHGTEVk | 0.53 | 173.5712 | 5 | 3908.885 | 2.967 |
|  |  | vSPIcTMEyVPHcGSDGVTySNR | 0.52 | 143.187 | 4 | 3061.460 | 1.992 |
|  |  | YPSTVSk | 40.32 | 0.013201 | 2 | 925.519 | 8.739 |
|  |  | tLVAcPR | 27.86 | 0.357726 | 3 | 960.545 | 3.763 |
|  |  | yPSTVSk | 22.95 | 0.783047 | 3 | 1213.716 | 0.800 |
|  |  | tSTLkDGR | 24.64 | 0.680932 | 2 | 1165.682 | 3.323 |
|  |  | eYDGEcRPk | 18.24 | 1.99758 | 3 | 1441.709 | 7.520 |
|  |  | eEVPELDcSk | 17.93 | 3.136732 | 2 | 1493.756 | 10.978 |
|  |  | lHDGEck | 9.36 | 13.81263 | 3 | 1146.579 | -2.222 |
| 440923751 | Ovalbumin | eVVGxAEAGVDAASVSEEFR | 119.99 | 2.03468E-10 | 2 | 2153.059618 | 5.4032927 |
|  |  | GGLEPINFQTAADQAR | 114.48 | 6.04006E-10 | 2 | 1687.832933 | -4.1342712 |
|  |  | gGLEPINFQTAADQAR | 99.57 | 3.18361E-08 | 2 | 1831.952196 | 5.579696 |
|  |  | iSQAVHAAHAEINEAGR | 97.44 | 5.68131E-08 | 2 | 1918.000414 | -0.4173567 |
|  |  | ISQAVHAAHAEINEAGR | 92.22 | 1.40591E-07 | 2 | 1773.906298 | 4.0285661 |
|  |  | LTEWTSSNVMEER | 90.52 | 4.85274E-08 | 2 | 1581.722704 | 0.8108556 |
|  |  | dILNQITKPNDVYSFSLASR | 90.26 | 3.15627E-07 | 3 | 2425.288303 | 1.5952311 |
|  |  | lPGFGDxIEAQcGTSVNVHSSLR | 87.89 | 4.10695E-07 | 4 | 2575.269918 | 0.2717645 |
|  |  | lTEWTSSNVMEER | 86.82 | 2.91781E-07 | 2 | 1725.832323 | 5.1214068 |
|  |  | dILNQITkPNDVYSFSLASR | 78.17 | 4.11647E-06 | 2 | 2569.405077 | 7.2310885 |
|  |  | aFKDEDTQAMPFR | 72.69 | 8.07943E-06 | 3 | 1699.823337 | 0.1371172 |
|  |  | vASMASEKMk | 71.78 | 1.69653E-05 | 2 | 1369.748705 | 4.7289881 |
|  |  | EVVGxAEAGVDAASVSEEFR | 70.34 | 1.23863E-05 | 2 | 2008.954882 | 4.4601714 |
|  |  | LTEWTSSNVmEER | 68.56 | 5.57959E-06 | 2 | 1597.715258 | -0.6751768 |
|  |  | eLINSWVESQTNGIIR | 68.45 | 4.32955E-05 | 2 | 2003.071093 | 1.5966586 |
|  |  | eVVGxAEAGVDAASVSEEFR | 67.55 | 3.07549E-05 | 3 | 2167.038547 | -11.576612 |
|  |  | AFKDEDTQAmPFR | 60.81 | 4.4729E-05 | 2 | 1571.722216 | 3.9824261 |
|  |  | lTEWTSSNVmEER | 60.67 | 8.13757E-05 | 3 | 1741.827915 | 5.4630608 |
|  |  | vASMASEk | 59.81 | 0.000187945 | 2 | 1110.613085 | 5.6972209 |
|  |  | aDHPFLFcIk | 59.77 | 0.000253 | 2 | 1535.835253 | 4.5130992 |
|  |  | VASMASEk | 57.99 | 0.000215725 | 2 | 966.507677 | 3.0860523 |
|  |  | VASmASEk | 57.86 | 0.000163354 | 2 | 982.4950427 | -4.6479462 |
|  |  | VVRFDKLPGFGDxIEAQcGTSVNVHSSLR | 57.81 | 0.000333886 | 3 | 3189.547458 | -19.954816 |
|  |  | aFkDEDTQAMPFR | 56.51 | 0.000513387 | 2 | 1843.933641 | 4.5956365 |
|  |  | ADHPFLFcIk | 55.82 | 0.000504131 | 2 | 1391.722582 | -2.6417058 |
|  |  | aFKDEDTQAmPFR | 55.15 | 0.000318323 | 3 | 1715.816685 | -0.7779135 |
|  |  | yPILPEYLQcVk | 54.81 | 0.00089811 | 2 | 1811.011156 | 5.2869344 |
|  |  | eVVGxAEAGVDAASVSEEFR | 54.05 | 0.000838852 | 3 | 2252.095981 | 0.3139822 |
|  |  | vASMASEkMk | 53.79 | 0.001058155 | 2 | 1513.849413 | 3.3837798 |
|  |  | vTEQESkPVQMmYQIGLFR | 53.69 | 0.001525331 | 4 | 2588.357809 | 4.7236483 |
|  |  | xGSIGAASMEFcFDVFk | 52.22 | 0.000907184 | 2 | 2096.010301 | 17.372627 |
|  |  | VTEQESkPVQMMYQIGLFR | 51.84 | 0.00220776 | 3 | 2428.265964 | 7.1491461 |
|  |  | yPILPEYLQcVk | 50.04 | 0.002261574 | 2 | 1955.111742 | 4.1417759 |
|  |  | dILNQITkPNDVySFSLASR | 49.67 | 0.002333222 | 2 | 2713.502245 | 5.0431373 |
|  |  | aFkDEDTQAmPFR | 46.6 | 0.003998134 | 2 | 1859.923632 | 1.9082951 |
|  |  | dEDTQAMPFR | 46.35 | 0.001573511 | 2 | 1353.629808 | 5.3294046 |
|  |  | YPILPEYLQcVk | 45.82 | 0.007808731 | 2 | 1666.905809 | 3.774069 |
|  |  | vASmASEk | 44.52 | 0.005673888 | 2 | 1126.606859 | 4.6039327 |
|  |  | YPILPEYLQcVK | 42.47 | 0.014323023 | 2 | 1522.791918 | -3.636468 |
|  |  | vTEQESkPVQmmYQIGLFR | 41.86 | 0.021852358 | 2 | 2604.343553 | 1.173485 |
|  |  | dEDTQAmPFR | 41.45 | 0.002313143 | 3 | 1369.613498 | -2.9284117 |
|  |  | eVVGxAEAGVDAASVSEEFR | 40.37 | 0.020281375 | 2 | 2137.058641 | 2.6050051 |
|  |  | gLWEkAFKDEDTQAMPFR | 38.07 | 0.050108422 | 4 | 2457.254781 | 2.923648 |
|  |  | IkVYLPR | 35.21 | 0.043357157 | 3 | 1032.670505 | 1.8021661 |
|  |  | DEDTQAmPFR | 34.86 | 0.004751853 | 2 | 1225.522386 | 5.6630267 |
|  |  | eVVGxAEAGVDAASVSEEFR | 32.89 | 0.116996335 | 2 | 2180.079637 | 9.5194424 |
|  |  | eLYRGGLEPINFQTAADQAR | 32.14 | 0.22329931 | 3 | 2393.25272 | 8.2044821 |
|  |  | eVVGxAEAGVDAASVSEEFR | 30.69 | 0.145154984 | 3 | 2226.079807 | 14.913572 |
|  |  | VTEQESkPVQmMYQIGLFR | 27.03 | 0.67639425 | 3 | 2444.259495 | 6.5358451 |
|  |  | hIATNAVLFFGR | 26.83 | 0.537402601 | 3 | 1489.848881 | 6.1927676 |
|  |  | vTEQESkPVQMmyQIGLFR | 24.63 | 1.150473119 | 4 | 2732.444235 | -1.2482329 |
|  |  | eVVGxAEAGVDAASVSEEFR | 22.22 | 1.272756664 | 3 | 2179.074253 | -11.826764 |
|  |  | vTEQESkPVQMMYQIGLFR | 15.04 | 10.97746654 | 2 | 2572.374803 | 9.382831 |
|  |  | gLWEkAFkDEDTQAmPFR | 8.38 | 49.77838625 | 4 | 2617.36025 | 5.9888195 |
|  |  | vASmASEKMk | 7.71 | 34.39505735 | 3 | 1385.737858 | 0.516215 |
|  |  | vTEQESkPVQmmyQIGLFR | 7.22 | 64.30781426 | 4 | 2748.441061 | -0.545602 |
|  |  | EVVGxAEAGVDAASVSEEFRADHPFLFcIk | 2.11 | 150.5645396 | 5 | 3351.650215 | 1.920391 |
|  |  | lPGFGDxIEAQcGTSVNVHSSLRDILNQITkPNDVYSFSLASR | 1.47 | 182.4547331 | 3 | 5054.542576 | 1.6105031 |
|  |  | vTEQESkPVQMMyQIGLFR | 0.74 | 271.3007916 | 4 | 2716.477682 | 9.1852545 |
|  |  | EVVGxAEAGVDAASVSEEFR | 27.7 | 0.251594797 | 2 | 1992.955492 | 2.2482362 |
|  |  | tQINKVVR | 19.11 | 1.350796874 | 3 | 1101.686985 | 0.8083384 |
|  |  | TQINKVVR | 35.76 | 0.029651944 | 2 | 957.5844592 | 0.4468743 |
|  |  | HIATNAVLFFGR | 23.87 | 1.011358216 | 2 | 1345.745043 | 5.537019 |
|  |  | vASmASEkMk | 21.81 | 1.892158666 | 2 | 1529.846727 | 4.9168234 |
|  |  | lTEWTSSNVmEERK | 10.29 | 19.95220303 | 2 | 1869.927294 | 7.4464887 |
|  |  | vASmASEKmk | 19.44 | 2.184813202 | 3 | 1401.731479 | -0.4122296 |
|  |  | xGSIGAASmEFcFDVFk | 24.32 | 0.444718386 | 3 | 2111.999424 | 14.498347 |
|  |  | ADHPFLFcIK | 17.79 | 2.385333741 | 2 | 1247.631761 | 6.0644223 |
|  |  | lYAEER | 22.59 | 0.728993986 | 2 | 924.492052 | 1.425316 |
|  |  | tQINKVVR | 19.11 | 1.350796874 | 3 | 1101.686985 | 0.8083384 |
|  |  | tQINk | 32.2 | 0.122138828 | 2 | 891.5538806 | 4.1313337 |
|  |  | gLWEk | 20.74 | 1.911839896 | 2 | 920.5431384 | -1.3675415 |
