# Supplementary material for: Proteomic comparison by iTRAQ combined with mass spectrometry of egg white proteins in laying hens (Gallus gallus) fed with soybean meal and cottonseed meal
Source: PLoS One. 2017 Aug 15;12(8):e0182886. doi: 10.1371/journal.pone.0182886 (PMC5557595; doi:10.1371/journal.pone.0182886)
Supplement: S1 Fig — (DOCX) [file pone.0182886.s003.docx]

**Supporting information**


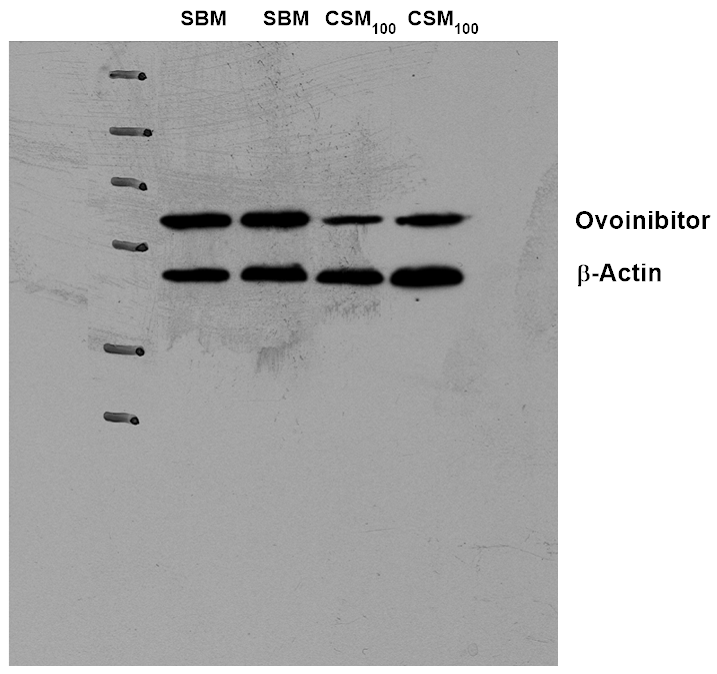


**S1 Fig. The western-blot of Ovoinhibitor expressed in SBM and CSM_100_**


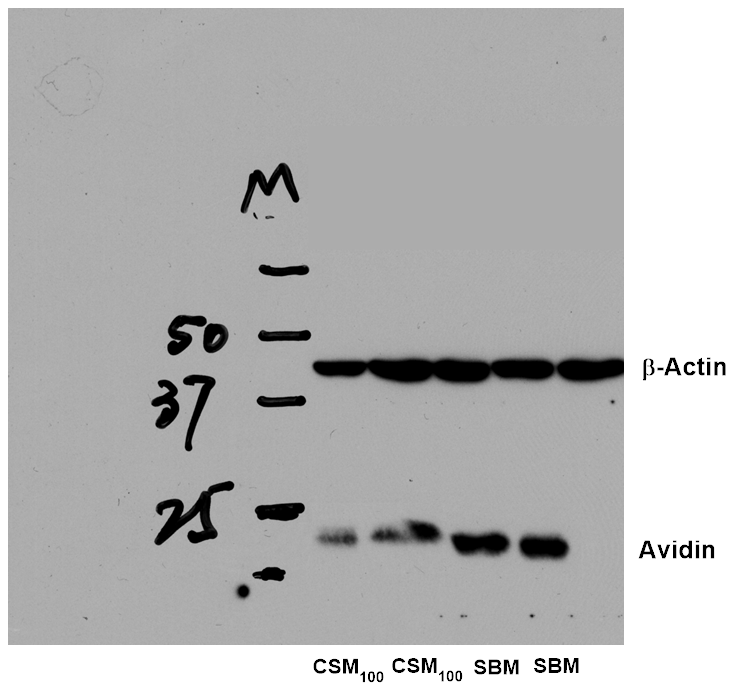


**S1 Fig. The western-blot of Avidin expressed in SBM and CSM_100_**


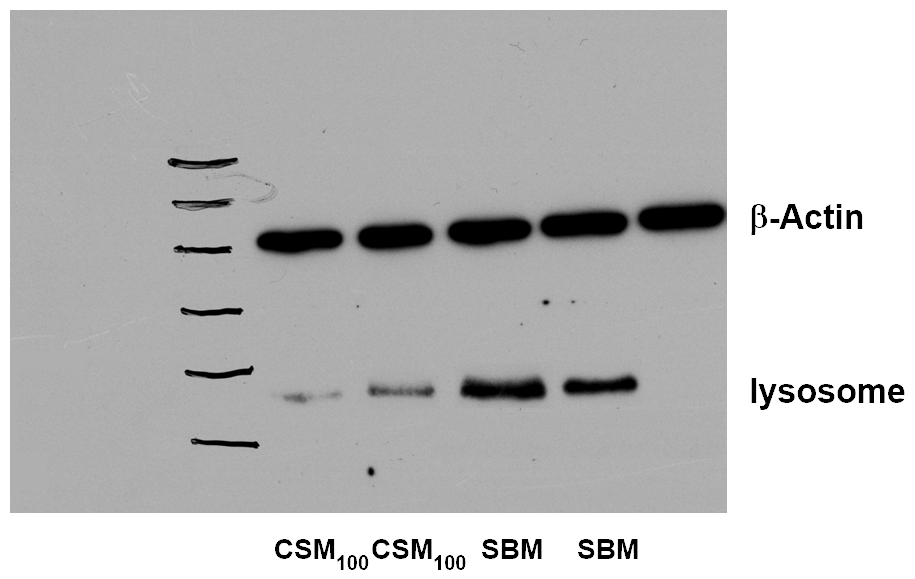


**S1 Fig. The western-blot of lysosome expressed in SBM and CSM_100_**
